# *Pseudomonas syringae* infectivity correlates to altered transcript and metabolite levels of* Arabidopsis* mediator mutants

**DOI:** 10.1038/s41598-024-57192-x

**Published:** 2024-03-21

**Authors:** Jeanette Blomberg, Viktor Tasselius, Alexander Vergara, Fazeelat Karamat, Qari Muhammad Imran, Åsa Strand, Martin Rosvall, Stefan Björklund

**Affiliations:** 1 Department of Medical Biochemistry and Biophysics, Umeå University, 901 87 Umeå, Sweden; 2 Department of Physics, Umeå University, 901 87 Umeå, Sweden; 3 Department of Plant Physiology, Umeå Plant Science Centre, Umeå University, 901 87 Umeå, Sweden; 4 Present Address: Biostatistics, School of Public Health and Community Medicine, Gothenburg University, P.O. Box 463, 405 30 Gothenburg, Sweden

**Keywords:** Plant sciences, Plant cell biology, Plant immunity, Plant molecular biology, Plant physiology, Plant stress responses, Secondary metabolism, Transcriptomics, Transcriptional regulatory elements

## Abstract

Rapid metabolic responses to pathogens are essential for plant survival and depend on numerous transcription factors. Mediator is the major transcriptional co-regulator for integration and transmission of signals from transcriptional regulators to RNA polymerase II. Using four Arabidopsis Mediator mutants, *med16*, *med18*, *med25* and *cdk8*, we studied how differences in regulation of their transcript and metabolite levels correlate to their responses to *Pseudomonas syringae* infection. We found that *med16* and *cdk8* were susceptible, while *med25* showed increased resistance. Glucosinolate, phytoalexin and carbohydrate levels were reduced already before infection in *med16* and *cdk8*, but increased in *med25*, which also displayed increased benzenoids levels. Early after infection, wild type plants showed reduced glucosinolate and nucleoside levels, but increases in amino acids, benzenoids, oxylipins and the phytoalexin camalexin. The Mediator mutants showed altered levels of these metabolites and in regulation of genes encoding key enzymes for their metabolism. At later stage, mutants displayed defective levels of specific amino acids, carbohydrates, lipids and jasmonates which correlated to their infection response phenotypes. Our results reveal that *MED16*, *MED25* and *CDK8* are required for a proper, coordinated transcriptional response of genes which encode enzymes involved in important metabolic pathways for Arabidopsis responses to *Pseudomonas syringae* infections.

## Introduction

Mediator is an important eukaryotic transcriptional co-regulator required for transmission of signals from promoter-bound transcriptional regulators to RNA polymerase II (Pol II)^1,2^. Mediator is composed of three core modules; Head, Middle and Tail^3^, and a fourth more loosely associated, regulatory Kinase module^4^. Tail subunits are main receivers of signals from transcriptional regulators, while Middle transfer signals from Tail to Head which makes direct contact with Pol II. Plant and human comprise more Mediator subunits than yeast, but its overall structure is conserved^5^. Mediator subunits MED8, MED15, MED16, MED18, MED20, MED25 and CDK8 were identified as important for diverse types of infection responses^6–11^. Each subunit has specific functions and can show either positive or negative effects on expression of specific genes, depending on the infection type. Most reports on Mediator subunit functions in immune responses have focused on their transcriptional effects. Only few describe how they affect responses at the metabolite level.

Pathogen attack is sensed by innate plant immune receptors present on the host cell surfaces or in their cytoplasm. Host surface receptor binding of microbial antigens called pathogen-associated molecular patterns (PAMPs) induces PAMP-triggered immunity (PTI)^12^, while recognition of pathogen derived effectors by intracellular nucleotide-binding/leucine-rich-repeat (NLR) receptors induces an effector-triggered immune (ETI) response^13^. Signaling cascades from PTI and ETI receptors lead to transcriptional reprogramming of several genes and constitute the base for a defense system producing numerous metabolites.

Salicylic acid (SA) and jasmonic acid (JA) are well-studied hormones that activate distinct defense pathways. SA plays key roles in defense against hemibiotrophs and biotrophs, such as *P. Syringae*, whereas JA acts with ethylene to induce resistance against necrotrophic pathogens. These pathways negatively regulate each other. Biosynthesis of SA during infection is mainly promoted by induced *ISOCHORISMATE SYNTHASE1 (ICS1)* expression, which in turn is positively regulated by *ENHANCED DISEASE SENSITIVE 1 (EDS1)* and *PHYTOALEXIN-DEFICIENT4 (PAD4)*. Signaling downstream from SA is executed by activation of *NONEXPRESSOR OF PATHOGENESIS-RELATED GENES1 (NPR1)* which translocates from the cytoplasm to the nucleus where it induces expression of various defense genes, for example *NIMIN1* and pathogen regulated (PR) genes. Reprogramed transcription of defense genes induces a long-term memory which is distributed throughout the plant, termed systemic acquired resistance (SAR). Thus, production of SA and signaling from SA is critical for functional PTI, ETI, and SAR responses^14^.

Plants also alter production of numerous secondary metabolites in response to pathogen attack. Glucosinolates (GLSs) constitute a large family of sulfur- and nitrogen-containing β-d-thioglucoside-*N*-hydroxysulfate defense compounds. Three classes are present in Arabidopsis: methionine-derived aliphatic GLSs, phenylalanine-derived benzenic GLSs, and tryptophan-derived indolic GLSs. The most abundant foliar GLSs are derived from methionine or tryptophan by chain elongation, core structure formation and secondary modifications. These GLSs are biologically inactive but are rapidly hydrolyzed and liberated from the glucose moiety by myrosinases upon pathogen attack. This transforms them into active compounds, such as isothiocyanates (ITCs), thiocyanates, simple nitriles and epithionitriles. Transcription Factors (TFs) of the MYB R2R3-family control GLS biosynthesis by activation of genes encoding key enzymes in these pathways. MYB28, MYB29 and MYB76 regulate expression of *MAM1*, *CYP79F1* and *CYPF2* which encode key enzymes in the aliphatic pathways^15^, while MYB34, MYB51 and MYB122 regulates *CYP79B2* and *CYP79B3*, which encode key enzymes in the indolic pathway. Besides indolic GLSs, other indolic phytoalexins contributing to the defense response, like camalexin, indole-3-carbaldehyde (ICHO), indole carboxylic acid (ICOOH) and ascorbigen, are also produced from tryptophan^16^.

Lipids (fatty acids, oxylipins, phospholipids, glycolipids, glycerolipids, sphingolipids, and sterols) also influence plant-pathogen interactions at various levels. Oxylipins are derived from polyunsaturated fatty acids and are formed enzymatically or non-enzymatically. The enzymatic pathway starts with lipoxygenases and leads to formation of 12-oxophytodienoic acid (OPDA), jasmonates, aldehydes and other oxylipin metabolites such as monogalactosyldiacylglycerols (MGDGs) and digalactosyldiacylglycerols (DGDGs). Several complex lipid molecular species where fatty acids are linked to OPDA or dinor-OPDA (dnOPDA) have been characterized^17–19^. Here we combine metabolic profiling and RNA-seq to reveal mechanisms for transcriptional and metabolite responses to *P. syringae* infection in wild type Arabidopsis and Mediator subunit mutants.

## Results

### *med16* and *cdk8* show increased, and *med25* reduced susceptibility to *P. syringae* infection

**Figure 1:**
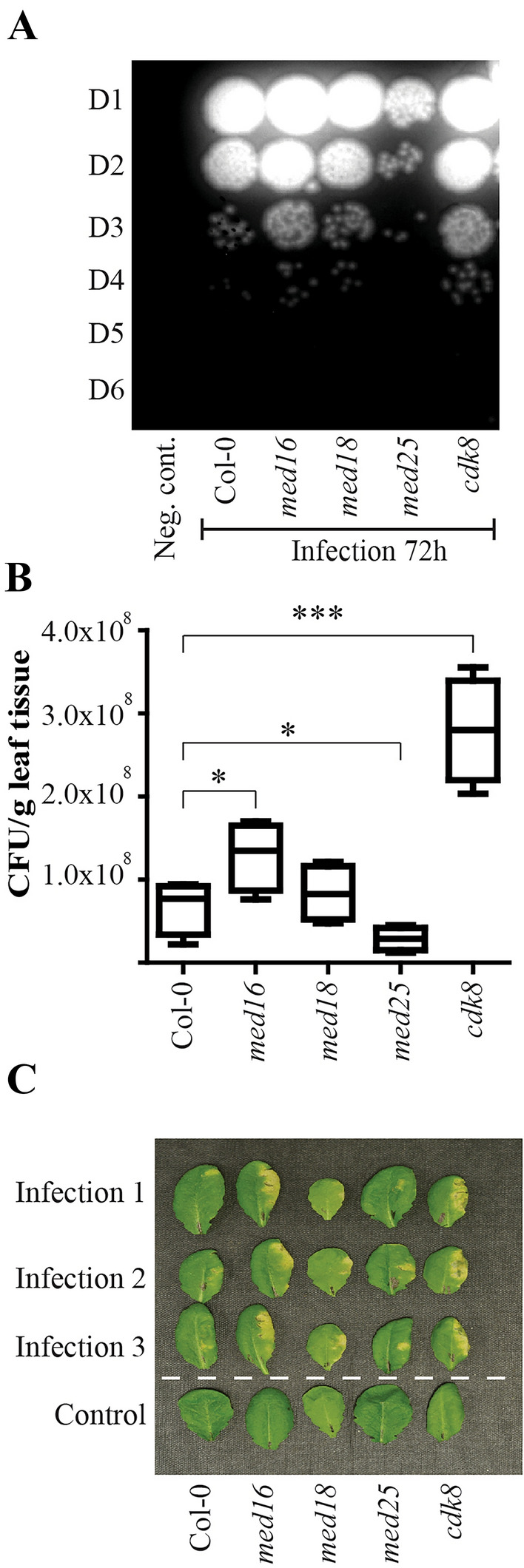
Susceptibility of Col-0 and Mediator mutants to infection by *P. syringae*. Four leaves each of 5-week-old plants were injected with either 10 mM MgCl_2_ (control) or 1 × 10^6^ CFU/ml of the virulent *Pst.* DC3000. (**A**) Bacteria were extracted from 100 mg of grounded tissue from mock-treated and infected plants and colonies formed from 10 × serial dilutions (D1 to D6) at the 72-h time point are shown. (**B**) The box plot represents the means of CFUs of the D3 dilution from four independent infections. Asterisks indicate significant differences between each mutant and Col-0 (Student’s *t* test, *p < 0.05; **p < 0.01; ***p < 0.001) and the error bars show mean standard deviation (SD) of four independently infected plants. (**C**) An illustrative picture of disease symptoms visible as necrotic and chlorotic lesions on leaf surfaces from three independently infected plants (infection 1–3) and one control experiment.

*Med16*, *med18*, *med25*, and *cdk8* mutants, representing each of the tail, middle, head, and kinase Mediator modules, display diverse defects in defense signaling in response to different infections^6,7,9,20,21^. To investigate their responses to *P. syringae*, we infected Col-0 and mutants with *P. syringae* pv. *tomato* DC3000, a virulent strain that suppresses PTI by injecting effectors into cells via bacterial secretion systems. This generates a mild disease response that becomes visible after 2–3 days in the form of wet, chlorotic, and spreading necrotic lesions^22^. Mediator mutants also display flowering-time phenotypes^23–25^. To avoid developmental effects, we infected our plants under non-inductive, short-day conditions at the age of 5–6 weeks. Colony-forming units (CFUs) for each line was determined 72 h post infection (p.i.) using serial dilution (Fig. 1A, B). *med16* and *cdk8* showed significantly higher, and *med25* lower CFUs relative to Col-0. A statistically non-significant increase was also detected in *med18* in accordance with other reports^26^. Susceptibility of *med16* and *cdk8* was also detected as more severe disease symptoms of leaves at 72 h (Fig. 1C).

### Global metabolite levels in Col-0 and mutants deviate between infected and mock-treated leaves with the highest differences detected 72 h p.i.

**Figure 2:**
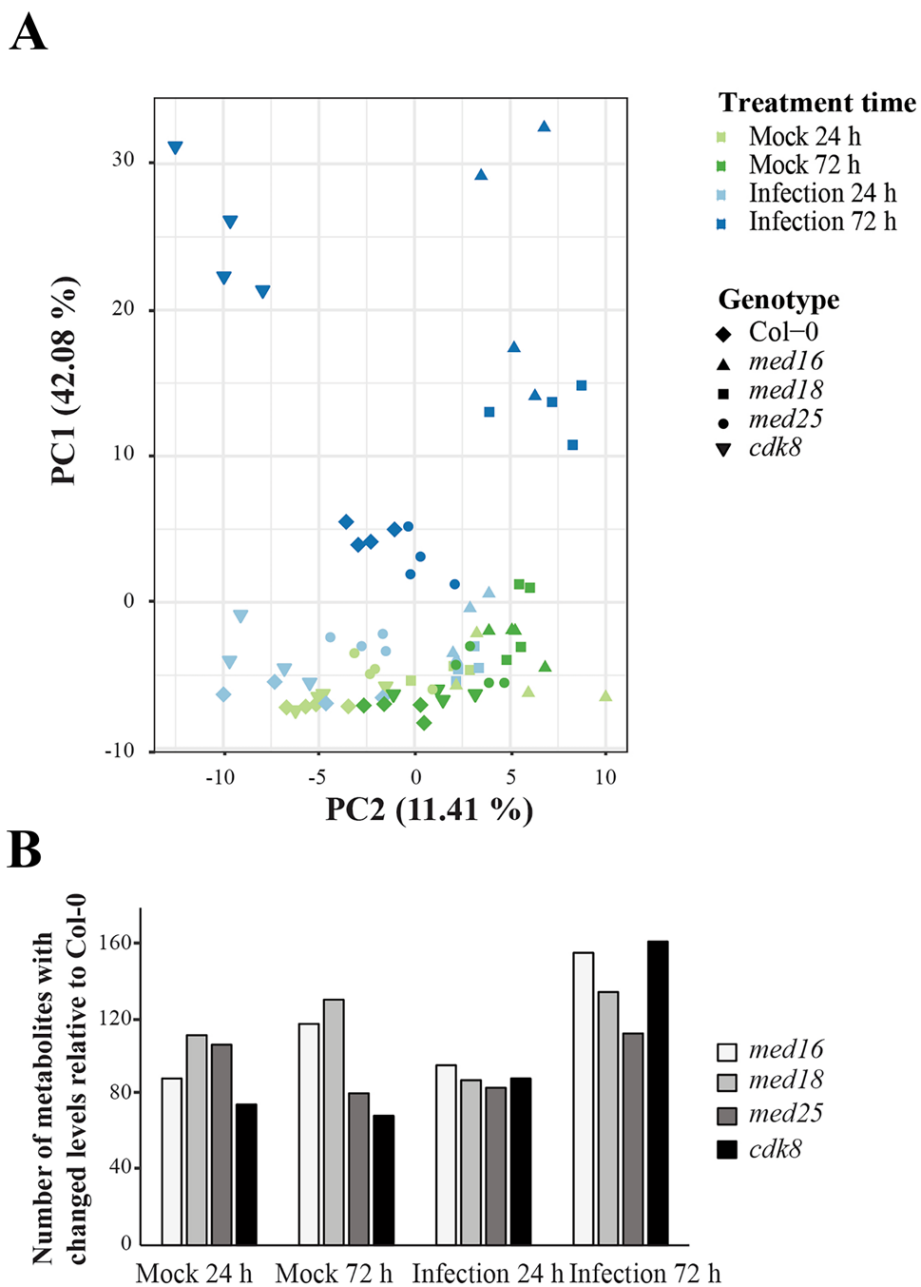
Non-targeted metabolomic profiling of Col-0 and Mediator mutants at the 24- and 72-h time points after infection. (**A**) Multivariate statistical analysis of GC–MS and LC–MS non-targeted metabolite profiles of infected and mock-treated Col-0, *med16*, *med18*, *med25* and *cdk8* leaves at the 24- and 72-h time points are shown as a PCA score plot. Each data point represents the entire metabolome (GC–MS plus LC–MS) for each replicate and are arranged in the first and second dimensions (x and y respectively). (**B**) Number of metabolites with statistically significant (Student's *t* test, *p* < 0.05) altered levels in mutants compared to Col-0 at the indicated time points and treatments.

Untargeted LC–MS and GC–MS were performed at 24 and 72 h p.i. to determine global metabolite levels in infected and mock-treated Col-0 and mutants. We recorded 171 and 75 metabolites from the LC–MS and GC–MS, respectively (Supplementary Table 1). For twenty-four metabolites identified in both assays, we decided to use the LC–MS data resulting 222 metabolites used for further analyses. An overview of metabolite levels in Col-0 and mutants at both timepoints after infection or mock-treatment (Control) is illustrated as a principal component analysis (PCA; Fig. 2A). PC1 and PC2 accounted for 42.08% and 11.41% of the total variation, respectively. The profiles in mutants differed slightly from Col-0 under control conditions (PC2) while major differences were detected after infection, especially at 72 h (PC1). The four independent infection replicates for each mutant and treatment grouped well, indicating high reproducibility.

In agreement with their increased susceptibility, *med16* and *cdk8* showed the largest number of changed metabolite levels at both 24 and 72 h after infection (Fig. 2B). Lists of normalized peak values of all metabolites and their ratios in infected and mock-treated Col-0 and mutants at each time point are shown (Supplementary Tables 1–2). Differences were further visualized using heatmaps and hierarchical clustering (Supplementary Fig. 1) and confirm that the sensitive *med16* and *cdk8* mutants display pronounced increases of secondary metabolites at 72 h, in particular sugars, lipids, and amino acids.

### Uninfected Col-0 and mutants display differences in metabolite levels of specific pathways

**Figure 3:**
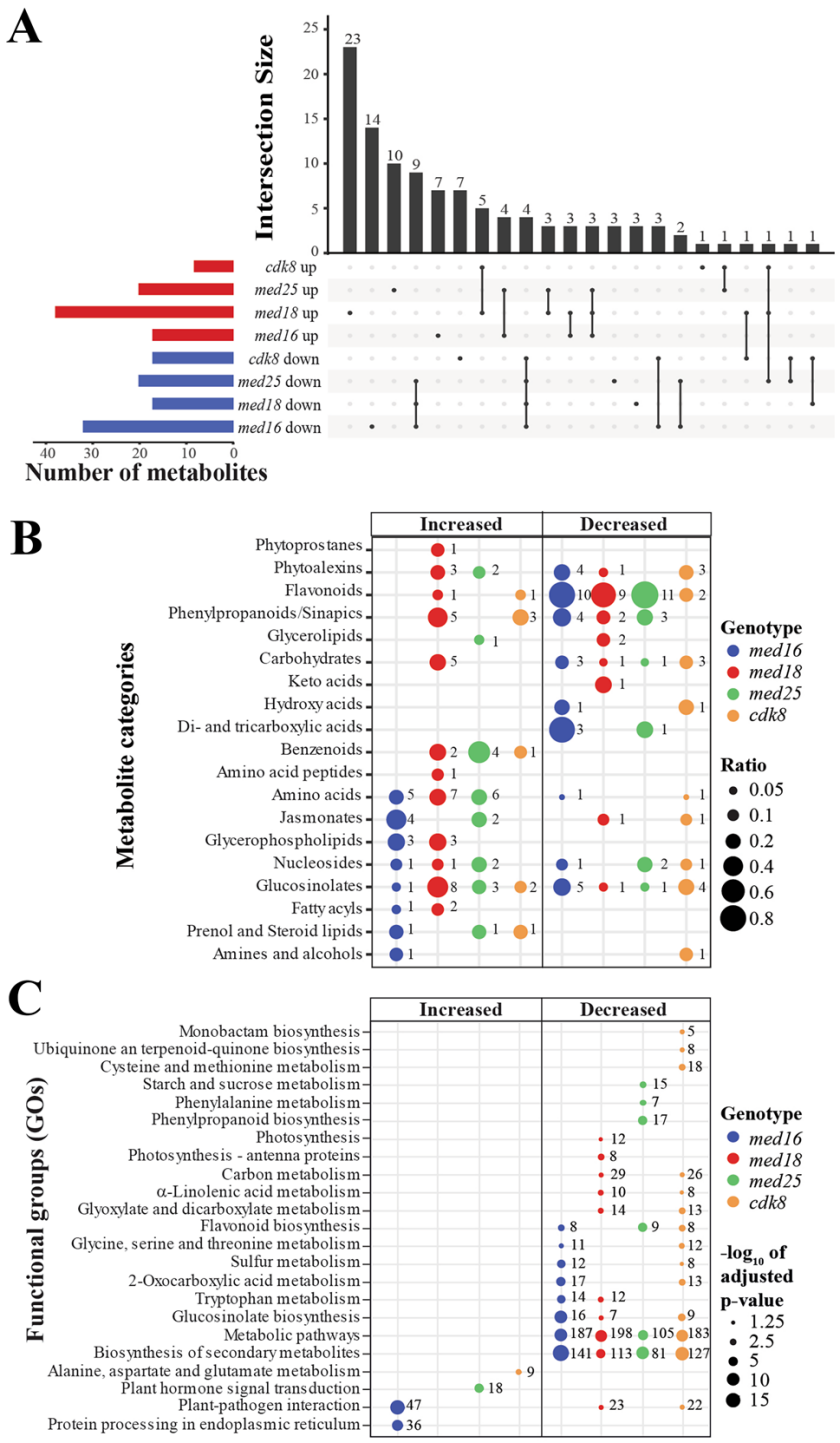
Comparison of metabolites and mRNAs with altered levels in Mediator mutants relative to Col-0 in mock-treated plants. (**A**) Upset diagram illustrating both the overlap and uniqueness of metabolites that display altered levels in mock-treated mutants relative to Col-0. Only metabolites that show statistically significant differences (log_2_-FC > 0.5 or < − 0.5 and *p* < 0.05) at both the 24- and 72-h time points are included. The total number of metabolites that display altered levels in each mutant are represented as red (increased) and blue (decreased) bars to the left. (**B**) Metabolites that displayed statistically distinct levels in mock-treated mutants relative to mock-treated Col-0 were grouped into nineteen metabolite categories (see Supplementary Table 3). Sizes of circles represent the ratio of the number of metabolites in each category that display significantly different levels in mutants relative to Col-0 and the total amount of metabolites detected for each category in our full data set of 222 metabolites. Numbers to the right of each circle represent the total number of metabolites that show significantly changed levels in each mutant. (**C**) GO enrichment analysis of alterations in defined KEGG-pathways^50–52^ of differentially expressed genes (DEGs) in each mutant relative to Col-0. The sizes of circles represent the significance (-log_10_ of Benjamini–Hochberg adjusted p-value) of the enrichment for each GO and numbers to the right of circles represent the number of DEGs for each mutant in the respective GO.

To identify metabolites that differ between mock-treated Col-0 and mutants, those that displayed a significant difference at both time points were identified. *med16* showed the highest number of decreased and *med18* the highest number of increased metabolites (Fig. 3A). To illustrate these differences comprehensively, we grouped them into nineteen categories according to their classification in the Human Metabolome Database (https://hmdb.ca/; Supplementary Table 3). The mutants displayed significant differences in levels of metabolite categories relative to Col-0 even in mock-treated lines (Fig. 3B). Phytoalexins, carbohydrates and GLSs were reduced in the susceptible *med16* and *cdk8* but increased in *med18* and/or the resistant *med25*. In support, RNA-seq showed that *med16* and *cdk8* displayed a pronounced reduction of transcripts encoding GLS biosynthesis enzymes (Fig. 3C, Supplementary Table 4). We also noticed reduction in *med16* and *cdk8* of transcripts belonging GO categories “Glycine, serine and threonine metabolism”, “Sulfur metabolism” and “2-oxocarboxylic acid metabolism”, which represent pathways linked to GLS metabolism. Finally, *med16*, *med18* and *cdk8* showed defects in expression of “plant-pathogen interactions” genes, albeit in different directions where *med18* and *cdk8* showed decreased, while *med16* showed increased levels.

Since *med16* and *cdk8* are susceptible, while *med25* shows increased resistance to *P. syringae*, we focused on metabolites that displayed similar differences in *med16* and *cdk8* relative to Col-0 in mock-treated plants, and those that where uniquely different from Col-0 in each of *med16*, *med25* and *cdk8* at both timepoints. No metabolites were commonly increased in *med16* and *cdk8* relative to Col-0, but two GLSs (Sulforaphane and a Sulforaphane fragment) and one phytoalexin (Benzyl dithiocarbamate) were decreased and might contribute to susceptibility (Supplementary Table 5). To obtain a broader view of GLS levels in mutants, we calculated their mean in mock-treated mutants relative to Col-0 at the 24- and 72-h time points. Five GLSs (Sulforaphane, a Sulforaphane fragment, Hirsutin, a Hirsutin fragment and Neoglucobrassicin) displayed reduced levels in the sensitive *med16* and *cdk8* (Supplementary Fig. 2A). In contrast, GLS levels were increased in *med18* and *med25*. We also detected several phytoalexins that were reduced in *med16* and *cdk8* but increased in *med18* and *med25* (Supplementary Fig. 2B).

Fourteen metabolites were uniquely decreased in mock-treated *med16* and eight in *cdk8* at both time points (Supplementary Table 5). They represent various metabolite categories, but five (*med16*) and three (*cdk8*) were GLSs or phytoalexins. Using mean values at 24- and 72-h, we identified five (*med16*) and six (*cdk8*) downregulated GLSs, and four (*med16*) and three (*cdk8*) downregulated phytoalexins (Supplementary Fig. 2A, B). In line with the opposite phenotype of *med25* versus *med16* and *cdk8*, GLSs and phytoalexins were upregulated in *med25*. None of the uniquely decreased metabolites in *med25*, and none of the uniquely increased metabolites in *med16* and *cdk8* were GLSs or phytoalexins (Supplementary Table 5). Thus, GLS and phytoalexin levels in mock-treated mutants correlate with their *P. syringae* susceptibility. Finally, ten metabolites were uniquely increased in *med25*. The most common were benzenoids (Supplementary Fig. 2C) which are derivatives of SA.

### Reduction of metabolite levels during the early infection response is impaired in *med16* and *med18*

**Figure 4:**
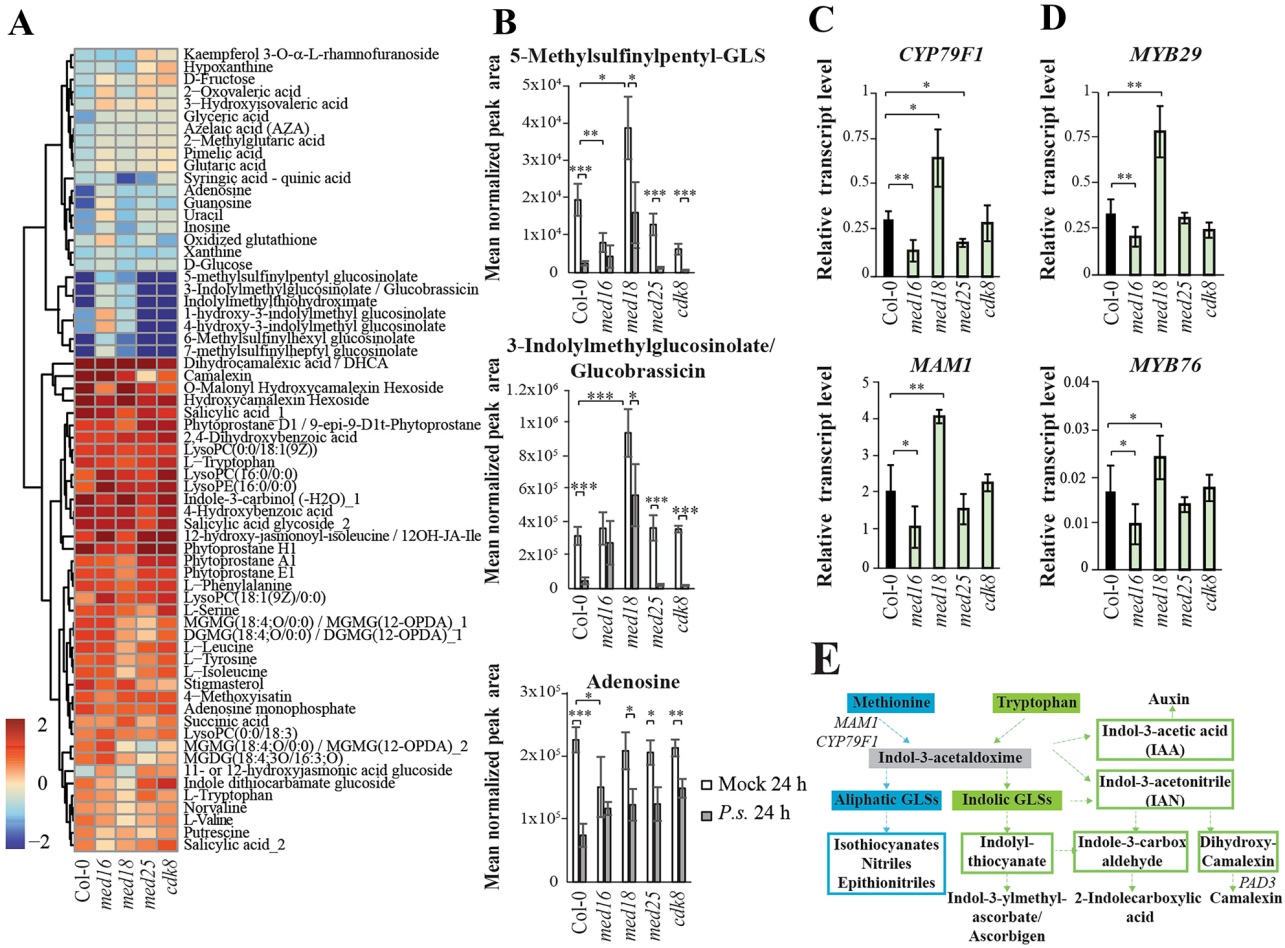
Impaired reduction of specific metabolite levels is caused by different mechanisms in *med16* and *med18*. (**A**) Heat map and hierarchical clustering of metabolites that show a log_2_-FC > 0.5 or < − 0.5, and *p* < 0.05 in infected relative to mock-treated Col-0 at the 24-h time point. (**B**) Bar graphs of normalized mean peak areas from four independently infected or mock-treated Col-0 and mutant plants at the 24-h time point. (**C**) Quantification of transcript levels for two of the aliphatic-GSL biosynthesis enzymes (*CYP79F1* and *MAM1*) in uninfected Col-0 and mutants using RT-qPCR. (**D**) Quantification of transcript levels for two of the aliphatic-GSL biosynthesis regulating transcription factors (*MYB29* and *MYB76*) in uninfected Col-0 (black bars) and mutants (light green bars) using RT-qPCR. Asterisks in B-D indicate pairwise significant differences (Student's *t* test, *p < 0.05; **p < 0.01; ***p < 0.001) for comparisons indicated by the brackets. Error bars represent the SD of four independent infection replicates for metabolite levels and three replicates for transcript levels. (**E**) Simplified overview of the aliphatic-GLS, indolic-GLS and phytoalexin synthetic pathways. Each arrow indicates multiple enzymatic steps and only the enzymes analyzed here are indicated.

Hierarchical clustering of all metabolites displaying changed levels in Col-0 at 24 h relative to its control revealed a set of decreased GLSs and nucleosides, whereas phytoprostanes, lipids, amino acids, benzenoids, jasmonates, and phytoalexins were increased (Fig. 4A, Supplementary Tables 6–7). Mock-treated plants were used as controls rather than untreated to avoid unwanted wounding responses elicited by mechanical manipulation. The reduction of GLSs in Col-0 was absent in *med16* and impaired in *med18*. Similarly, the reduction in nucleosides at the early timepoint in Col-0, was absent in *med16* and defect in the other mutants (Supplementary Table 6). Lack of metabolite reduction in mutants can result from different mechanisms. One possibility is that levels are reduced even in mock-treated mutants, and remain low after infection. Alternatively, mutants can have normal levels before infection but are unable to reduce them in response to infection. To distinguish between these possibilities, we compared the absolute levels for one representative of each GLS class: 5-methylsulfinylpentyl-GLS (aliphatic) and 3-Indolylmethylglucosinolate/Glucobrassicin (indolic), and one nucleoside (adenosine). The mechanisms for the lack of metabolite reduction differed between mutants and metabolites (Fig. 4B). *med16* showed dysregulation of aliphatic GLSs and nucleosides at 24 h due to reduced levels in mock-treated controls. In contrast, *med16* was deficient in Glucobrassicin reduction in response to infection. Mock-treated *med18* exhibited elevated levels of both aliphatic and indolic GLSs, as we have reported^27^. This results in inadequate reduction of these metabolites in *med18* which after infection displayed GLSs levels similar to those observed in mock-treated Col-0 (Fig. 4B). Metabolites showing significant fold changes in each mutant, are shown (Supplementary Tables 6–7). For the full set of log_2_FCs of all metabolites in all mutants relative to Col-0, see Supplementary Table 2.

We used RT-qPCR to quantify mRNA levels encoding key enzymes in the aliphatic GLS pathway in mock-treated Col-0 and mutants (Fig. 4C). *CYP79F1* and *MAM1* mRNA levels were increased in *med18* but decreased in *med16*, correlating with their aliphatic GLS levels (Fig. 4B). MYB28, MYB29 and MYB76 belong to the R2R3-family of MYB TFs which regulate expression of *CYP79F1* and *MAM1*^15^. In line with the effects observed for their target genes, *MYB29* and *MYB76* mRNA levels were decreased in mock-treated *med16* but increased in *med18* (Fig. 4D). For an overview of the GLS and phytoalexin metabolic pathways, see Fig. 4E.

### Metabolites induced in Col-0 during the early response show diverse types of impaired induction in mutants

**Figure 5:**
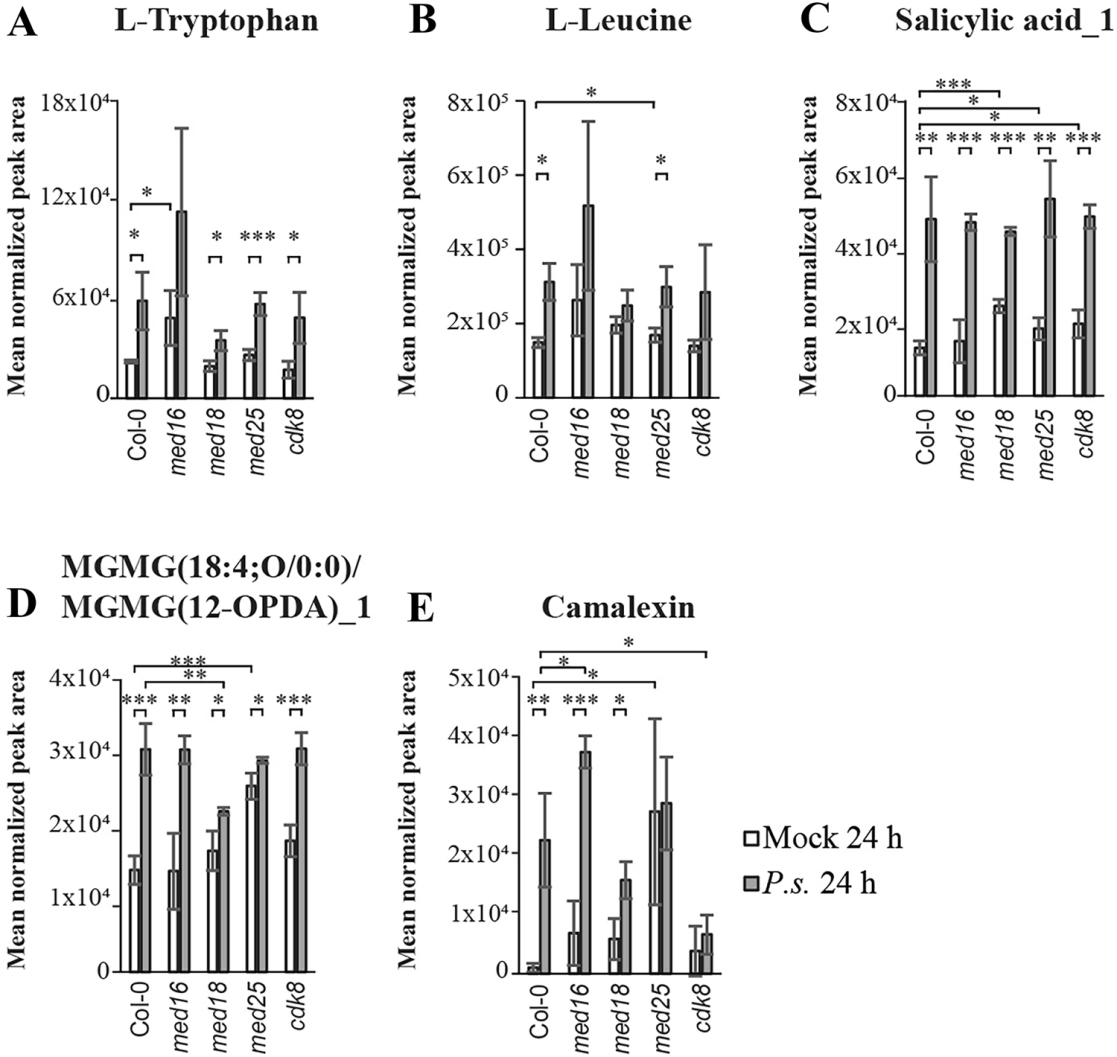
All mediator mutants have unique alterations in the induction of specific metabolites compared to Col-0 at the early time point after infection. Metabolite levels of L-Tryptophan (**A**), L-Leucine (**B**), Salicylic acid_1 (**C**), MGMG (18:4;O/0:0) (**D**), and camalexin (**E**) in infected and mock-treated Col-0 and mutants at the 24-h time point. Asterisks indicate pairwise significant differences between Col-0 and each mutant, or between mock-treated and infected lines as indicated by the brackets (Student's *t* test, *p < 0.05; **p < 0.01; ***p < 0.001) and the error bars show the SD of four independently infected plants.

All significantly increased metabolites in Col-0 at 24 h relative to control, and their levels in mutants are shown in the heat map (Fig. 4A). Metabolites that showed a log_2_FC > 1 in Col-0, and their fold changes in each mutant are listed (Supplementary Table 7). Representative metabolites that were increased at 24 h in Col-0 but showed defect levels in one or several mutants are shown (Fig. 5A–E).

A set of amino acids were increased in Col-0 at 24 h (Supplementary Table 7). In contrast, tryptophan showed no significant induction in *med16* and reduced induction in *med18* (Fig. 5A). Lack of tryptophan-induction in *med16* was mainly caused by elevated levels in mock-treated cells, while *med18* was unable to induce tryptophan to the same extent as Col-0 in response to infection (Fig. 5A). Furthermore, the elevated leucine levels observed in Col-0 were missing in *med16*, *med18* and *cdk8* (Fig. 5B).

Four benzenoids were induced in Col-0 at 24 h (Supplementary Table 7). One was SA, which also showed induced levels in all mutants. However, we detected increased absolute SA-levels in mock-treated *med18*, *med25* and *cdk8* (Fig. 5C). SA is produced by hydroxylation at the second position of the benzene ring of benzoic acid and can also be named 2-OH-benzoic acid. Additional hydroxylation at different benzene ring positions creates different benzenoids such as 4-OH-bensoic acid and 2,4-di-OH-benzoic acid. Conjugation of the hydroxylated benzoic ring to a glucose moiety results in formation of the hydroxy-benzoic acid glucosides SAG (SA-glucoside) and SGE (SA-glucose ester) which causes translocation into different cellular compartments^28^. We identified two different glycosylated forms of SA (SA-glycoside_1 (SAG_1) and SA-glycoside_2 (SAG_2) in our LCMS-assays. (Supplementary Fig. 3A-B). SAG_1 was present at low levels in Col-0 and did not increase upon infection, while SAG_2 was highly induced. Interestingly, SAG_2 levels were increased in *med25.* Even though Col-0 showed a higher fold-induction of SAG_2 (3.3-fold) compared to *med25* (2.0-fold) at 24 h, the absolute levels were the same in mock-treated *med25* as in infected Col-0 (Supplementary Fig. 3B). Similar elevated levels in mock-treated *med25* were also found for three other metabolites that represent modified versions of benzoic acid: 4-hydroxybenzoic acid, 2,4-dihydroxybenzoic acid and Protocatechuic acid 3-glucoside (Supplementary Fig. 3C–E), suggesting that elevated levels of several SA-related metabolites in *med25* before infection might contribute to its resistance against *P. syringae*.

ICS1 is the key enzyme for pathogen-induced SA production (Supplementary Fig. 4A). We found that the fold induction of *ICS1* mRNA was significantly higher in *med25* relative to the other *Pst*-infected lines at 24 h. (Supplementary Fig. 4B). Furthermore, expression levels of *EDS1* mRNA, encoding one of the transcriptional activators of *ICS1* expression, were increased in *Pst*-infected *med25* relative to Col-0, *med16* and *med18* at 24 h, while it was reduced in *cdk8* (Supplementary Fig. 4C). Interestingly, EDS1 physically interacts with CDK8^29^. In contrast, the other *ICS1* transcriptional activator *PAD4* did not show significant differences in any mutant. Responses downstream of SA are mediated by NPR1, a transcriptional co-activator for SA-dependent genes (*NIMIN1* and *PR1*). We found that *NIMIN1* and *PR1* transcript levels were similar in *med25* and Col-0 but impaired in *med16*, *med18* and *cdk8* (Supplementary Fig. 4D).

Levels of oxylipins with conjugated OPDA (e.g. MGMG(18:4;O/0:0)/MGMG(12-OPDA)_1 and DGMG(18:4;O/0:0)/DGMG(12-OPDA)_1) were clearly induced in Col-0, *med16* and *cdk8* (Fig. 5D, Supplementary Table 7). In contrast, they were less induced in *med18* and *med25*, albeit by different mechanisms. Levels were similar in mock-treated *med18* and Col-0, but uninduced in *med18*. In contrast, mock-treated *med25* displayed levels equivalent to Col-0 at 24 h but showed no increase after infection. As for the SA-related metabolites, increased levels of OPDA-conjugated oxylipins in uninfected *med25* might contribute to its resistance.

Camalexin and a set of related compounds were the most highly induced metabolites in Col-0. (Fig. 5E, Supplementary Table 7). However, camalexin induction was impaired in *med25* and *cdk8*. Again, these differences were due to different mechanisms. In mock-treated *med25*, camalexin was elevated to even higher levels than Col-0 after infection. In contrast, mock-treated *cdk8* displayed low levels but no increase at 24 h. This might contribute to their opposite phenotypes. The difference is likely due to defects in *PAD3* expression, which encodes the key enzyme in the camalexin synthesis pathway (Supplementary Fig. 5A). *PAD3* expression was higher in uninfected *med25* but lower in *cdk8* relative to Col-0 (Supplementary Fig. 5B). Finally, induction of some phytoprostanes and AMP was slightly defect in *med16* and *med18* (Supplementary Table 7).

**Table 1:** Summary of linear regression estimates of log_2_-fold changes for mutants relative to Col-0.

Mutant	Slope estimate	Adjusted R^2^	*p* value
*med16*	0.826	0.5518	0.0006
*med18*	1.024	0.6231	0.648
*med25*	1.819	0.7194	< 2e−16
*cdk8*	0.729	0.8548	< 2e−16

### Sugars, amino acids and jasmonates accumulate in *med16* and *cdk8* but are decreased in *med25* at the late stage of infection

**Figure 6:**
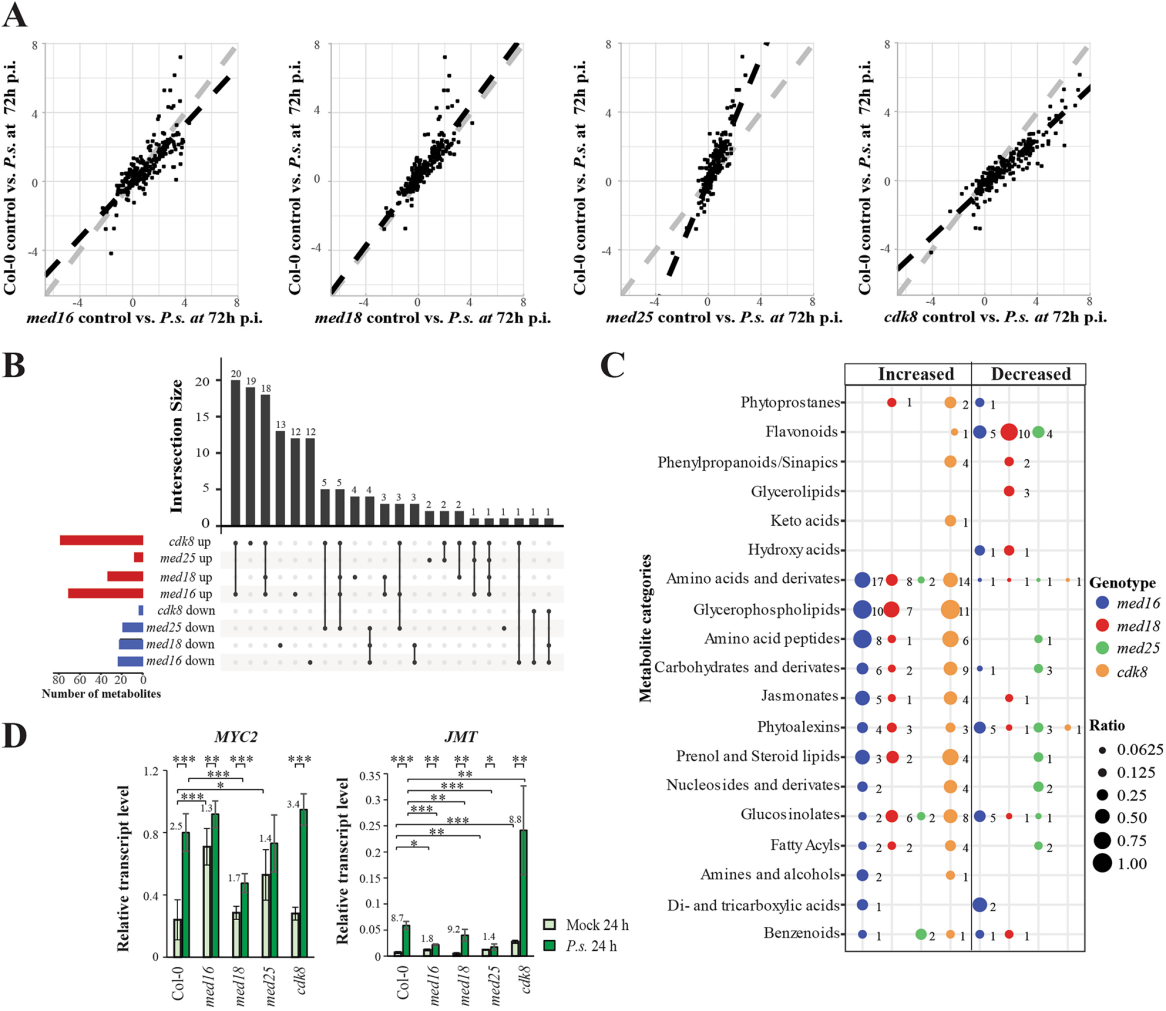
*med25* shows an attenuated infection response whereas *med16* and *cdk8* show the highest alterations in metabolite levels compared to Col-0 at the late stage of infection. (**A**) Scatter plots of the relationship between metabolite changes at 72 h p.i. (relative to control at 72 h) between each mutant (x-axis) and Col-0 (y-axis). (**B**) Upset diagram illustrating the overlap and uniqueness in increased and decreased metabolite levels in each mutant relative to Col-0 at 72 h p.i. Total number of metabolites displaying changed levels in each mutant are represented as red (increased) and blue (decreased) bars to the left. (**C**) Distribution of metabolites displaying changed levels in each mutant for each of the nineteen metabolite categories. Sizes of circles represent the ratio of the of the number of metabolites in each category that display significantly distinct levels in mutants relative to Col-0 and the total amount of metabolites detected for each category in our full data set of 222 metabolites. Numbers to the right of each circle represent the total number of metabolites that show significantly changed levels in each mutant. In (**A**) a significance cutoff of log_2_-FC > 0.5 or < − 0.5 and *p* < 0.05) was used. In (**B**, **C**) a significance cutoff of log_2_-FC > 1 or < − 1 and *p* < 0.05) was used. The experiments were performed using four independently infected plants. (**D**) Levels of two JA activated genes (*MYC2* and *JMT*) in mock-treated and infected cells at the 24-h time point. Asterisks indicate pairwise significant differences between mock-treated and infected lines as indicated by the brackets (Student's *t* test, *p < 0.05; **p < 0.01; ***p < 0.001) and the error bars show the SD of three independently infected plants.

We made scatterplots representing changes in levels of all identified metabolites at 72 h relative to control. The distribution of log_2_-FCs was compared between each mutant and Col-0 using linear regression (Fig. 6A). To reveal deviations in mutants, we tested the null hypothesis that the slope of the linear regression was equal to one. Table 1 shows slope estimates and adjusted R^2 for the regression as well as *p* values for significance of deviation from slope equal one. *med16* and *cdk8* had significantly lower slopes indicating that their metabolic responses were more affected relative to Col-0, while the *med25* slope indicates that it on average was less affected. The slope for *med18* was similar to Col-0. These results correlate with the phenotypic differences between mutants*.*

For metabolites that were increased in mutants, we found a large overlap of 48 metabolites between *med16* and *cdk8* (Fig. 6B). Twenty-four of them were also increased in *med18* (Supplementary Table 8). In line with its opposite phenotype only three were increased, while eight were reduced in *med25*. Using a less strict threshold we identified a set of amino acids (leucine, phenylalanine, 5-hydroxy-L-tryptophan, and tyrosine) which were increased in *med16* and *cdk8* but decreased in *med25* (Supplementary Fig. 6A). The fold change for aspartic acid showed the reverse, being reduced in *med16*, and *cdk8* but increased in *med25*. We also identified twenty-five lipids that where commonly increased in *med16* and *cdk8*. One of them, the unsaturated lipid stigmasterol, was reduced in *med25.* Thus, stigmasterol levels correlate to bacterial growth in each mutant. Finally, twelve carbohydrates showed increased fold change in *med16* and/or *cdk8*, and half of them were reduced in *med25*. In particular, trehalose showed the highest fold increase compared to Col-0 in *cdk8* (log_2_FC = 3.7) but was decreased in *med25* (log_2_FC = − 1.4).

The sensitive *med16* and *cdk8* mutants displayed increased jasmonate levels after infection (Fig. 6C). *med16* showed accumulation of 12-hydroxylated-JA-Ile, *cdk8* displayed pronounced increase of methylated JA (MEJA), while both mutants showed induced levels of the active form of JA (JA-Ile) (Supplementary Fig. 6B). α-linolenic acid (18:3), a fatty acid substrate of jasmonate biosynthesis, was also elevated in the *med16* and *cdk8* (Supplementary Fig. 6B), suggesting a broad and dysfunctional regulation of the jasmonate biosynthetic pathway in these mutants. The increased levels of jasmonates in *med16* corroborated its increased mRNA levels of JA-related genes relative to the other mutants in mock-treated plants (Supplementary Fig. 6C).

Many JA-induced genes are regulated by MYC2. We found that mRNA levels of *MYC2* in both *med16* and *med25* under control conditions were elevated relative to those detected in Col-0. However, induction of *MYC2* was reduced in *med16* and *med25*, resulting in similar levels of *MYC2* in *Pst*-infected Col-0, *med16*, *med25* and *cdk8* at 24 h (Fig. 6D). MED25 is important for MYC2-dependent gene regulation^20^. Accordingly, induction of *JMT*, a MYC2 target gene encoding an S-adenosyl-L-methionine:JA carboxyl methyltransferase that catalyzes formation of MEJA from JA, was severely reduced in *med16* and *med25* due to a combination of elevated levels before, and deficient induction after infection (Fig. 6D). *cdk8* displayed elevated levels of *JMT* before infection but in contrast to *med16* and *med25*, it showed the same fold-induction of *JMT* as Col-0. Thus, elevated MEJA levels could result from increased expression of *JMT* in *cdk8*. Two other MYC2 target genes, *JAZ6* and *LOX2*, were uninduced in *med25* after infection (Supplementary Fig. 6D). The same impairment was detected for *med16* after infection whereas *cdk8* showed accumulation of *JAZ6*.

## Discussion

Mediator plays important roles for biotic and abiotic stress responses. Mutations in *MED2*, *MED14*, *MED16*, *MED18*, and *MED25* causes impaired responses to drought, high salinity and cold^30,31^, and MED8, MED14, MED15, MED16, MED18, MED20, and MED25 are involved in responses to biotic stress^6,10,32^. Furthermore, Mediator subunits are important for the SA and JA/Ethylene signaling pathways, which control several aspects of defense signaling in plants^8,26,33^. Interactions between Mediator subunits and regulatory TFs are important for responses to biotrophic pathogens such as *P. syringae*^34,35^. Less is known regarding how Mediator mutations affect metabolite levels and how such changes influence infection susceptibility. We show that *med16* and *cdk8* are susceptible to *P. syringae* while *med25* shows increased resistance compared to Col-0. We identify dysregulation of specific metabolites that can explain the phenotypic variations and show that differences in metabolite responses between Col-0 and mutants can result from pre-existing variations already in uninfected plants, or in how their levels change in response to infection.

In uninfected cells, three metabolite categories showed differences in responses in mutants relative to Col-0 which might explain the variations in phenotypes for each of *med16*, *med25* and *cdk8*. Phytoalexins, carbohydrates and GLSs were reduced in the susceptible *med16* and *cdk8* but were increased in the more resistant *med25*. This agrees with previous reports showing that the levels of these metabolites are induced in Col-0 in response to infections^36–38^. In particular, GLSs are known to increase in infected Col-0 and to play important roles in infection defense^39^. We found reduced levels of Sulforaphane and derivatives in uninfected, susceptible *med16* and *cdk8* but increased levels in the resistant *med25*. Sulforaphane is produced in response to several non-host bacterial pathogens, and *P. syringae* strains adapted to Arabidopsis express *SURVIVAL IN ARABIDOPSIS EXTRACTS* (*SAX*) genes, which enable bacteria to detoxify host-produced sulforaphane^40^. We also identified two additional GLSs (Hirsutin and Neoglucobrassicin) that showed reduced levels in uninfected *med16* and *cdk8* and additional GLSs, carbohydrates and phytoalexins that were uniquely reduced in *med16* or *cdk8*. In line with our metabolite profiling of uninfected Col-0 and mutants, our RNA-seq showed that *med16* and *cdk8* have reduced levels of transcripts encoding enzymes involved in GLS biosynthesis and metabolism and expression of *PAD3* which encodes a key enzyme in synthesis of camalexin was increased in uninfected *med25* but lower in *cdk8*. Finally, a set of metabolites were uniquely elevated in uninfected *med25*. The most common category was benzenoids, which are precursors for SA synthesis. We conclude that levels of GLSs and phytoalexins in mock-treated mutants correlate with their susceptibility to *P. syringae* infection. Since SA plays a major role in defense against *P. syringae*^41^, the elevated levels of several SA-related metabolites in *med25* already before infection might contribute to its resistance.

In the early response to *P. syringae*, we identified a further set of metabolites whose fold-change deviate in mutants. One important category was amino acids, which play important functions as precursors for several defense-mediating secondary metabolites. Tryptophan is an important precursor for camalexin, indolic GLSs, and auxin (indole-3-acetic acid; IAA) synthesis. Our results show that tryptophan before infection are increased in *med16* to the same levels as those we observe in Col-0 and the other mutants after infection. Phenylalanine, a precursor for flavonoids, anthocyanins, benzoic acids and SA, was induced in Col-0 and all mutants but its absolute levels were increased in *med16* both before, and at the early timepoint after infection. A set of benzoic acids, among them SA, showed dysregulation in the mutants at the early timepoint after infection. We identified two differently regulated glycosylated forms of SA. SAG_1 was present at low levels in both Col-0 and *med25*, while SAG_2 was similarly induced in Col-0 and *med25* but present in much higher levels in the mutant. These metabolites likely correspond to SA-glucoside (SAG) and SA-glucose ester (SGE), but were indistinguishable using LCMS. Based on previously reported results showing that the levels of SGE in Arabidopsis is lower than SAG and that SGE is much less induced in response to wounding compared to SAG^42^, it is likely that SGE in our assays corresponds to SAG_1 and SAG to SAG_2. Camalexin, and a set of modified Camalexin-related metabolites were uninduced in both *med25* and *cdk8* relative to Col-0. Again, the mechanisms for the lack of induction differed between mutants. Uninfected *med25* displayed Camalexin levels even higher than infected Col-0 and they did not increase further upon infection. In contrast, *cdk8* showed low Camalexin levels both before and after infection. Finally, oxylipins with conjugated OPDA were induced in all lines, but most prominently in Col-0, *med16* and *cdk8* at the early time point after infection.

At the late time point after infection, we observed several differences between Col-0 and mutants. Generally, *med16* and *cdk8* showed larger effects, while *med25* showed less. Nearly fifty metabolites were present at higher levels at the late time point in both *med16* and *cdk8* relative to Col-0 but were unaffected or present at lower levels in *med25*. Of these, a set of amino acids and twenty-five lipids were commonly increased in *med16*, and *cdk8*. One of them was stigmasterol, which was also reduced in *med25.* Stigmasterol is synthesized at pathogen inoculation sites where it integrates into plant cell membranes and favors susceptibility to bacterial pathogens^43^. Also, a set of carbohydrates showed increased levels in infected *med16* and *cdk8*, while they were decreased in *med25*. In particular, trehalose was highly increased in *cdk8* but severely decreased in *med25*. Application of exogenous trehalose was recently shown to significantly increase susceptibility to *P. syringae*^44^, suggesting that its elevated levels might contribute to the increased and decreased bacterial content in *cdk8* and *med25*, respectively. Finally, we identified increased levels of jasmonates in *med16* and *cdk8* at the late time point. Jasmonates comprise a family of oxylipins including JA that regulate various aspects of plant immunity and development. It is therefore likely that the dysregulation of JA we observe contributes to their phenotypic behavior in response to infection. One possible explanation to the increased levels of some metabolites could originate from the infecting bacteria, especially those that are common between bacteria and plants like amino acids, lipids and carbohydrates. However, our infection results show that *cdk8* at 72 h after infection contains twice as many bacteria compared to *med16* but we could not detect any clear difference for these metabolite categories between the two mutants (cf. Fig. 1B and Supplementary Fig. 6A).

Using a combination of metabolomics and RNA-seq and four Mediator mutants to study molecular mechanisms for how Arabidopsis respond to *P. syringae* infection, we identify a set of unique metabolites and metabolite categories that show different types of defects in mutants. These differences correlate with defects in regulation of genes encoding key enzymes or regulatory TFs that are important for a proper infection response. Our results form a base for future research that will hopefully result in plants and crops which are more resistant to infections.

## Methods

### Plant material and growth conditions

Wild type Columbia-0 (Col-0) *Arabidopsis thaliana* plants purchased from Nottingham Arabidopsis Stock Centre (NASC; https://arabidopsis.info/) were used as reference genotype for susceptibly to virulent *P. syringae* pv. *tomato* DC3000 (*Pst*. DC3000). Mediator mutants in the Col-0 background: *med16* (alias sfr6-2; SALK_048091), *med18* (SALK_027178), *med25* (SALK_129555) and *cdk8* (GABI_564F11), were purchased from Salk Institute genomic analysis laboratory (SALK; http://signal.salk.edu/) and Nottingham Arabidopsis Stock Centre (NASC; https://arabidopsis.info/) and have been described previously^27,45,46^. Plants were grown in soil under controlled short day (8 h light/16 h dark) conditions at 22 °C and 67% humidity. All plant experiments/protocols were performed with relevant institutional, national, and international guidelines and legislations.

### Bacterial infection and colony forming unit (CFU) assay

Single colonies were picked and grown in Kings Broth (KB) liquid media containing 50 µg/ml rifampicin at 28 °C over night. Cultures were diluted to OD_600_ of 0.3 and grown to an OD_600_ of 0.8–1. Exponentially growing bacteria was collected, washed twice in 10 mM MgCl_2_ and resuspended in the same solution at a concentration of 2 × 10^6^ CFU/ml. 4–6 lower leaves of 5–6-week-old plants were injected on their abaxial side with either *Pst.* DC3000 suspension (infections) or 10 mM MgCl_2_ (controls). Leaves from 2–3 plants were pooled for each biological replicate. For quantification of bacterial growth, 40 mg pooled leaf samples were grinded in 1 mL 10 mM MgCl_2_, serially diluted and plated on KB plates containing 50 µg/ml rifampicin. Bacterial numbers were determined using the following formula: CFU = ((Number of colonies × volume × dilution factor)/volume plated)/sample weight.

### Metabolite extraction

Samples were prepared from control and infected 5-week-old plants^47^. 20 mg of grinded samples were mixed with 1 mL extraction buffer (20/20/60 v/v/v chloroform:water:methanol) including internal standards for GC–MS and LC–MS. LC–MS internal standards were: 13C9-phenylalanine, 13C3-caffeine, D4-cholic acid, D8-arachidonic acid and 13C9-caffeic acid (Sigma, St. Louis, MO, USA). GC–MS internal standards were: L-proline-13C5, alpha-ketoglutarate-13C4, myristic acid-13C3, cholesterol-D7 (Cambridge Isotope Laboratories, Inc., Andover, MA, USA) and succinic acid-D4, salicylic acid-D6, L-glutamic acid-13C5,15N, putrescine-D4, hexadecenoic acid-13C4, D-glucose-13C6, D-sucrose-13C12 from Sigma. The samples were bead-beated and centrifuged as described^27^. Most of the supernatants, 200 µL for LC–MS analysis and 50 µL for GC–MS analysis, were transferred to micro vials, evaporated to dryness and stored at − 80 °C until analysis. Small aliquots of the remaining supernatants were pooled and used as quality control (QC) samples. MSMS analysis (LC–MS) was run on the QC samples for identification purposes. The samples were analyzed in batches according to a randomized run order on both GC–MS and LC–MS.

### GC–MS profiling

Derivatization and GC–MS analysis were performed as described^47^. 0.5 μL of the derivatized sample was injected in splitless mode by an L-PAL3 autosampler (CTC Analytics AG, Switzerland) into an Agilent 7890B gas chromatograph equipped with a 10 m × 0.18 mm fused silica capillary column with a chemically bonded 0.18 μm Rxi-5 Sil MS stationary phase (Restek Corporation, U.S.) The injector temperature was 270 °C, the purge flow rate was 20 mL/min, and the purge was turned on after 60 s. The gas flow rate through the column was 1 mL/min. The column temperature was held at 70 °C for 2 min, then increased by 40 °C/min to 320 °C and held there for 2 min. The column effluent was introduced into the ion source of a Pegasus BT time-of-flight (TOF) mass spectrometer, GC/TOFMS (Leco Corp., St Joseph, MI, USA). The transfer line and the ion source temperatures were 250 °C and 200 °C, respectively. Ions were generated by a 70-eV electron beam at an ionization current of 2.0 mA, and 30 spectra/s were recorded in the mass range m/z 50–800. The acceleration voltage was turned on after a solvent delay of 150 s. The detector voltage was 1800–2300 V.

### LC–MS profiling

The samples were reconstituted in 20 µL of 50% (v/v) methanol before analysis. Each batch of samples were initially analyzed in positive mode, followed by a switch to negative mode for a second injection of each sample after analyzing all samples within the batch. The chromatographic separation was performed on an Agilent 1290 Infinity UHPLC-system (Agilent Technologies, Waldbronn, Germany). Two μL of each sample were injected onto an Acquity UPLC HSS T3, 2.1 × 50 mm, 1.8 μm C18 column in combination with a 2.1 mm × 5 mm, 1.8 μm VanGuard precolumn (Waters Corporation, Milford, MA, USA) held at 40 °C. The gradient elution buffers were A (0.1% formic acid) and B (75/25 (vol/vol) acetonitrile:2-propanol, 0.1% formic acid), and the flowrate was 0.5 mL/min. The compounds were eluted with a linear gradient consisting of 0.1—10% B over 2 min. B was then increased to 99% over 5 min and held at 99% for 2 min. B was then decreased to 0.1% over 0.3 min and the flow-rate was increased to 0.8 mL/min for 30 s. These conditions were kept for 0.9 min, after which the flow-rate was reduced to 0.5 mL min^−1^ for 0.1 min before the next injection. The compounds were detected using an Agilent 6546 Q-TOF mass spectrometer equipped with a jet stream electrospray ion source operating in positive or negative ion mode. The settings were kept identical between the modes, with exception of the capillary voltage. A reference interface was connected for accurate mass measurements. The reference ions purine (4 μM) and HP-0921 (Hexakis(1H, 1H, 3H-tetrafluoropropoxy)-phosphazine) (1 μM) were infused directly into the MS (flow rate of 0.05 mL/min) for internal calibration, and the monitored ions were purine m/z 121.05 and m/z 119.03632; HP-0921 m/z 922.0098 and m/z 966.000725 for positive and negative mode respectively. The gas temperature was set to 150 °C, the drying gas flow to 8 L/min and the nebulizer pressure to 35 PSI. The sheath gas temperature was set to 350 °C and the sheath gas flow to 11 L/min. The capillary voltage was set to 4000 V in positive ion mode, and to 4000 V in negative ion mode. The nozzle voltage was 300 V. The fragmentor voltage was 120 V, the skimmer 65 V and the OCT 1 RF Vpp 750 V. The collision energy was set to 0 V. The m/z range was 70–1700, and data was collected in centroid mode with an acquisition rate of four scans/s (1977 transients/spectrum).

### Metabolite data analysis

For the GC–MS data, all non-processed MS-files from the metabolic analysis were exported from the ChromaTOF software in NetCDF format to MATLAB R2021a (MathWorks, Natick, MA, USA), where all data pre-treatment procedures, such as base-line correction, chromatogram alignment, data compression and Multivariate Curve Resolution were performed. The extracted mass spectra were identified by comparisons of their retention index and mass spectra with libraries of retention time indices and mass spectra^27^. Mass spectra and retention index comparison was performed using the NIST MS 2.2 software. Annotations of mass spectra were based on reverse and forward searches in the library. Masses and ratios between masses indicative of a derivatized metabolite were especially notified. The mass spectrum with the highest probability indicative of a metabolite and the retention index between the sample and library for the suggested metabolite was ± 5 (usually less than 3) and the deconvoluted “peak” was annotated as an identification of a metabolite. For the LC–MS data, all data processing was performed using the Agilent MassHunter Profinder version B.10.00 (Agilent Technologies Inc., Santa Clara, CA, USA). The processing was performed both in a targeted and an untargeted fashion. For target processing, a pre-defined list of metabolites was analyzed using the Batch Targeted feature extraction in MassHunter Profinder. An in-house LC–MS library built up by authentic standards run on the same system with the same chromatographic and mass-spec settings, was used for the targeted processing^27^. The identification of the metabolites was based on MS, MSMS and retention time information. Differences in metabolite concentrations were tested using pairwise *t* test assuming equal variance between groups. *p* values below 0.05 were considered significant and due to the exploratory nature of the metabolite analysis no correction for multiple testing was performed.

### RNA isolation and qPCR

Total RNA was extracted from 100 mg of grounded leaf tissue using the E.Z.N.A Plant Mini Kit (Omega Bio-tek, Norcross, USA) and contaminating DNA was removed using turbo DNAfree DNAse I (Ambion, Foster City, USA). Total RNA (1 µg) was reverse transcribed using iScript reverse transcription supermix (Biorad, Solna, Sweden). RT-qPCR was performed using a LightCycler 96 and the PowerUp SYBR green master mix (Applied Biosystems, Massachusetts, USA). Gene expression levels were normalized to the reference genes *RCE1* (AT4G36800) and *ACT2* (AT3G18780) and displayed as relative units. Three biological (independent infections) and two technical replicates were used for each sample. RT-qPCR sequence primers are shown in Supplementary Table 9.

### RNA-sequencing

RNA-seq data for uninfected Col-0, *med16*, *med18* and *cdk8* plants have been published^46^. RNA-seq data for uninfected *med25* and Col-0 were obtained in an equivalent manner. In brief, isolated and DNAse treated RNA extracted from 5-week-old plants was assayed for RNA integrity with the Agilent 2100 Bioanalyzer using the RNA Nano 6000 kit (Agilent Technologies, Santa Clara, USA). Single-end RNA-seq was performed on a HiSeq 2500 High Output V4 platform (Illumina, San Diego, USA), generating 13–32 million reads.

### Analysis of transcriptomic data

The raw RNA-seq data was pre-processed by NGI Uppsala using TrimGalore and FastQC. Reads were aligned to the Araport11 reference genome and read counts were obtained using the kallisto R package (v0.45.1)^48^. To analyze differential gene expression, the R DESeq2 package (v1.30.0)^49^ was employed. Using pairwise differential expression analysis comparisons, fold changes were obtained between the mutant genotypes and their respective wild-type controls (Supplementary Table 4). Thus, a comparison was made between mutants and wild-type plants under normal, non-stress conditions.

### Gene ontology (GO) functional enrichment analyses

GO functional enrichment analyses was done using Genomes (KEGG) pathway enrichment (www.ncifcrf.gov)^50–52^, with the whole genome background and a cutoff of an adjusted *p* value of 0.05 was applied to account for multiple hypothesis testing. Lists of enriched GO functional categories were reduced by removal of redundant categories using REVIGO (http://revigo.irb.hr) with default settings and the database set to “Arabidopsis thaliana”. Overlaps between gene sets were determined using http://bioinformatics.psb.ugent.be/webtools/Venn.

### Supplementary Information

Supplementary Information 1.

Supplementary Figure 1.

Supplementary Figure 2.

Supplementary Figure 3.

Supplementary Figure 4.

Supplementary Figure 5.

Supplementary Figure 6.

Supplementary Table 1.

Supplementary Table 2.

Supplementary Table 3.

Supplementary Table 4.

Supplementary Table 5.

Supplementary Table 6.

Supplementary Table 7.

Supplementary Table 8.

Supplementary Table 9.

## Data Availability

The sequencing data for the *med16*, *med18*, *cdk8* mutants and the wild type Col-0 has been deposited at the European Nucleotide Archive (ENA, www.ebi.ac.uk/ena) under accession number PRJEB33339. The sequencing data for the *med25* mutant and the wild type Col-0 has been deposited at https://figshare.com/s/9a58efd7fee99b1d755c. Similarly, The Metabolomics data have been deposited to the EMBL-EBI MetaboLights database (DOI: http://doi.org/10.1093/nar/gks1004. PubMed PMID: 23109552) with the identifier MTBLS8105. The complete dataset can be accessed here: https://www.ebi.ac.uk/metabolights/editor/MTBLS8105/descriptors.

## References

[1] Thompson CM, Koleske AJ, Chao DM, Young RA (1993). A multisubunit complex associated with the RNA polymerase II CTD and TATA-binding protein in yeast. Cell.

[2] Kim Y-J, Björklund S, Li Y, Sayre MH, Kornberg RD (1994). A multiprotein mediator of transcriptional activation and its interaction with the C-terminal repeat domain of RNA polymerase II. Cell.

[3] Dotson MR (2000). Structural organization of yeast and mammalian mediator complexes. Proc. Natl. Acad. Sci. U.S.A..

[4] Liao S-M (1995). A kinase–cyclin pair in the RNA polymerase II holoenzyme. Nature.

[5] Tsai K-L (2014). Subunit architecture and functional modular rearrangements of the transcriptional mediator complex. Cell.

[6] Kidd BN (2009). The mediator complex subunit PFT1 is a key regulator of jasmonate-dependent defense in *Arabidopsis*. Plant Cell.

[7] Wathugala DL (2012). The Mediator subunit SFR6/MED16 controls defence gene expression mediated by salicylic acid and jasmonate responsive pathways. New Phytol..

[8] Caillaud M-C (2013). A downy mildew effector attenuates salicylic acid-triggered immunity in Arabidopsis by interacting with the host mediator complex. PLoS Biol..

[9] Zhu Y (2014). CYCLIN-DEPENDENT KINASE8 differentially regulates plant immunity to fungal pathogens through kinase-dependent and -independent functions in *Arabidopsis*. Plant Cell.

[10] Fallath T (2017). MEDIATOR18 and MEDIATOR20 confer susceptibility to *Fusarium oxysporum* in *Arabidopsis thaliana*. PLoS ONE.

[11] Li X, Yang R, Chen H (2018). The *Arabidopsis thaliana* mediator subunit MED8 regulates plant immunity to *Botrytis Cinerea* through interacting with the basic helix-loop-helix (bHLH) transcription factor FAMA. PLoS ONE.

[12] Zhang J, Zhou J-M (2010). Plant immunity triggered by microbial molecular signatures. Mol. Plant.

[13] Jones JDG, Dangl JL (2006). The plant immune system. Nature.

[14] Durrant WE, Dong X (2004). Systemic acquired resistance. Annu. Rev. Phytopathol..

[15] Sønderby IE, Burow M, Rowe HC, Kliebenstein DJ, Halkier BA (2010). A complex interplay of three R2R3 MYB transcription factors determines the profile of aliphatic glucosinolates in *Arabidopsis*. Plant Physiol..

[16] Böttcher C (2014). The biosynthetic pathway of indole-3-carbaldehyde and indole-3-carboxylic acid derivatives in Arabidopsis. Plant Physiol..

[17] Stelmach BA (2001). A novel class of oxylipins, sn1-O-(12-oxophytodienoyl)-sn2-O-(hexadecatrienoyl)-monogalactosyl diglyceride, from *Arabidopsis thaliana*. J. Biol. Chem..

[18] Hisamatsu Y, Goto N, Sekiguchi M, Hasegawa K, Shigemori H (2005). Oxylipins Arabidopsides C and D from *Arabidopsis thaliana*. J. Nat. Prod..

[19] Kourtchenko O (2007). Oxo-phytodienoic acid-containing galactolipids in Arabidopsis: jasmonate signaling dependence. Plant Physiol..

[20] Chen R (2012). The *Arabidopsis* mediator subunit MED25 differentially regulates jasmonate and abscisic acid signaling through interacting with the MYC2 and ABI5 transcription factors. Plant Cell.

[21] Lai Z (2014). MED18 interaction with distinct transcription factors regulates multiple plant functions. Nat. Commun..

[22] Xin X-F, He SY (2013). *Pseudomonas syringae* pv. *tomato* DC3000: A model pathogen for probing disease susceptibility and hormone signaling in plants. Annu. Rev. Phytopathol..

[23] Cerdán PD, Chory J (2003). Regulation of flowering time by light quality. Nature.

[24] Knight H, Thomson AJW, McWatters HG (2008). Sensitive to freezing6 integrates cellular and environmental inputs to the plant circadian clock. Plant Physiol..

[25] Zheng Z, Guan H, Leal F, Grey PH, Oppenheimer DG (2013). Mediator subunit18 controls flowering time and floral organ identity in *Arabidopsis*. PLoS ONE.

[26] Zhang X, Wang C, Zhang Y, Sun Y, Mou Z (2012). The *Arabidopsis* mediator complex subunit16 positively regulates salicylate-mediated systemic acquired resistance and jasmonate/ethylene-induced defense pathways. Plant Cell.

[27] Davoine C (2017). Functional metabolomics as a tool to analyze Mediator function and structure in plants. PLoS ONE.

[28] Maruri-López I, Aviles-Baltazar NY, Buchala A, Serrano M (2019). Intra and extracellular journey of the phytohormone salicylic acid. Front. Plant Sci..

[29] Chen H (2021). Two interacting transcriptional coactivators cooperatively control plant immune responses. Sci. Adv..

[30] Bäckström S, Elfving N, Nilsson R, Wingsle G, Björklund S (2007). Purification of a plant mediator from *Arabidopsis thaliana* identifies PFT1 as the Med25 subunit. Mol. Cell.

[31] Hemsley PA (2014). The *Arabidopsis* mediator complex subunits MED16, MED14, and MED2 regulate mediator and RNA polymerase II recruitment to CBF-responsive cold-regulated genes. Plant Cell.

[32] Elfving N (2011). The *Arabidopsis thaliana* Med25 mediator subunit integrates environmental cues to control plant development. Proc. Natl. Acad. Sci. U.S.A..

[33] Dhawan R (2009). HISTONE MONOUBIQUITINATION1 interacts with a subunit of the mediator complex and regulates defense against necrotrophic fungal pathogens in *Arabidopsis*. Plant Cell.

[34] Seo JS, Diloknawarit P, Park BS, Chua N (2019). ELF18-INDUCED LONG NONCODING RNA 1 evicts fibrillarin from mediator subunit to enhance PATHOGENESIS-RELATED GENE 1 (PR1) expression. New Phytol..

[35] Huang J, Sun Y, Orduna AR, Jetter R, Li X (2019). The Mediator kinase module serves as a positive regulator of salicylic acid accumulation and systemic acquired resistance. Plant J..

[36] Tsuji J, Jackson EP, Gage DA, Hammerschmidt R, Somerville SC (1992). Phytoalexin accumulation in *Arabidopsis thaliana* during the hypersensitive reaction to *Pseudomonas syringae* pv *syringae*. Plant Physiol..

[37] Tierens KFM-J (2001). Study of the role of antimicrobial glucosinolate-derived isothiocyanates in resistance of Arabidopsis to microbial pathogens. Plant Physiol..

[38] Trouvelot S (2014). Carbohydrates in plant immunity and plant protection: Roles and potential application as foliar sprays. Front. Plant Sci..

[39] Andersson MX (2014). Involvement of the electrophilic isothiocyanate sulforaphane in Arabidopsis local defense responses. Plant Physiol..

[40] Fan J (2011). *Pseudomonas sax* genes overcome aliphatic isothiocyanate-mediated non-host resistance in *Arabidopsis*. Science.

[41] Vlot AC, Dempsey DA, Klessig DF (2009). Salicylic acid, a multifaceted hormone to combat disease. Annu. Rev. Phytopathol..

[42] Ogawa T, Ara T, Aoki K, Suzuki H, Shibata D (2010). Transient increase in salicylic acid and its glucose conjugates after wounding in Arabidopsis leaves. Plant Biotechnol..

[43] Griebel T, Zeier J (2010). A role for β-sitosterol to stigmasterol conversion in plant–pathogen interactions. Plant J..

[44] Wang X, Du Y, Yu D (2019). Trehalose phosphate synthase 5-dependent trehalose metabolism modulates basal defense responses in *Arabidopsis thaliana*. J. Integr. Plant Biol..

[45] Ng S (2013). Cyclin-dependent Kinase E1 (CDKE1) provides a cellular switch in plants between growth and stress responses. J. Biol. Chem..

[46] Crawford T (2020). Specific functions for mediator complex subunits from different modules in the transcriptional response of *Arabidopsis thaliana* to abiotic stress. Sci. Rep..

[47] Gullberg J, Jonsson P, Nordström A, Sjöström M, Moritz T (2004). Design of experiments: an efficient strategy to identify factors influencing extraction and derivatization of Arabidopsis thaliana samples in metabolomic studies with gas chromatography/mass spectrometry. Anal. Biochem..

[48] Bray NL, Pimentel H, Melsted P, Pachter L (2016). Near-optimal probabilistic RNA-seq quantification. Nat. Biotechnol..

[49] Love MI, Huber W, Anders S (2014). Moderated estimation of fold change and dispersion for RNA-seq data with DESeq2. Genome Biol..

[50] Kanehisa M (2000). KEGG: Kyoto encyclopedia of genes and genomes. Nucleic Acids Res..

[51] Kanehisa M (2019). Toward understanding the origin and evolution of cellular organisms. Protein Sci..

[52] Kanehisa M, Furumichi M, Sato Y, Kawashima M, Ishiguro-Watanabe M (2023). KEGG for taxonomy-based analysis of pathways and genomes. Nucleic Acids Res..

