# Supplementary material for: Pseudomonas syringae infectivity correlates to altered transcript and metabolite levels of Arabidopsis mediator mutants
Source: Sci Rep. 2024 Mar 21;14:6771. doi: 10.1038/s41598-024-57192-x (PMC10958028; doi:10.1038/s41598-024-57192-x)
Supplement: Supplementary file 1 — Supplementary Information 1. [file 41598_2024_57192_MOESM1_ESM.docx]

***Pseudomonas syringae* infectivity correlates to altered transcript and metabolite levels of *Arabidopsis* Mediator mutants**

Jeanette Blomberg^1^, Viktor Tasselius^2*^, Alexander Vergara^2^, Fazeelat Karamat^1^, Qari Muhammad Imran^1^, Åsa Strand^3^, Martin Rosvall^2^ and Stefan Björklund^1^

**SUPPLEMENTARY DATA**

**Supplementary Fig. 1. The two phenotypically most sensitive mutants, *med16* and *cdk8*, display the highest deviation of metabolite levels relative to Col-0 at 72 hours after *P. syringae* infection.** Heat map and hierarchical clustering of all 222 metabolites profiled after untargeted LC-MS and GC-MS analysis of mock-treated and *P. syringae-infected* Col-0, *med16*, *med18*, *med25* and *cdk8* at the 24- and 72-hour time points. Values represent average metabolite abundances scaled and centered to be comparable between metabolites.

**Supplementary Fig. 2. Mediator mutants display alterations in levels of glucosinolates, phytoalexins and benzenoids in uninfected plants which correlate to their phenotypes.** Log_2_-fold differences in levels of specific metabolites in *med16*, *med18*, *med25*, and *cdk8* relative to Col-0 at control conditions. Only differences with a p-value < 0.05 at both the 24- and 72-hour timepoints of mock-treated plants are included. Log_2_-fold differences in mutants relative to Col-0 for **(A)** Glucosinolates, **(B)** Phytoalexins and **(C)** Benzenoids at the 24-hour time point are shown. Dashed lines indicate a log_2_-fold difference of ±0.5.

**Supplementary Fig. 3. *med25* shows increased levels of benzoic acid derivates relative to Col-0 in both infected and mock-**treated **plants.** Metabolite levels of **(A)** Salicylic acid glycoside_1 (SGE), **(B)** Salicylic acid glycoside_2 (SAG), **(C)** 4-OH-Benzoic acid, **(D)** 2,4-di-OH-Benzoic acid, and **(E)** Protocatechuic acid-3-glucoside in infected and mock-treated Col-0 and *med25* at the 24- and 72-hour time points. Asterisks indicate pairwise significant differences between mock-treated Col-0 and *med25* and infected Col-0 and *med25* as indicated by the brackets (Student's t-test, *: p< 0.05, **: p< 0.01, ***: p< 0.001) and the error bars show mean standard deviation (SD) of four independently infected plants. Numbers without parentheses indicate fold change relative to mock-treated plants and numbers within parentheses indicate fold change of the comparisons indicated by the brackets.

**Supplementary Fig. 4. *med25* shows enhanced gene expression levels of the two SA signaling components *ICS1* and *EDS1* whereas *med16*, *med18* and *cdk8* show reduced expression of downstream salicylic acid activated target genes at 24 hours after infection. (A)** Simplified illustration of SA metabolic and signaling pathways activated by *P. syringae* infection. Relative real time qPCR of mRNA levels for **(B)**: the SA producing enzyme *ICS1*, **(C)**: the SA signaling activating factors: *EDS1* and *PAD4* and **(D)**: the SA signaling targets: *PR1* and *NIMIN1* in infected and mock-treated Col-0 and Mediator mutants at the 24-hour time point. Asterisks indicate pairwise significant differences between Col-0 and mutants at 24 hours after infection as indicated by the brackets (Student's t-test, *: p< 0.05, **: p< 0.01, ***: p< 0.001) and the error bars show +/- SD of three independently infected plants. Numbers above bars indicate fold change relative to mock-treated plants.

**Supplementary Fig. 5. *PAD3* expression is increased in *med25* but reduced in *cdk8*. (A)** Simplified illustrative picture of the function of PAD3 in camalexin synthesis. **(B)** Relative mRNA expression levels of *PAD3* in mock-treated Col-0 and Mediator mutants. Asterisks indicate pairwise significant differences between mock-treated Col-0 and each of the mock-treated *med25* and *cdk8* (Student's t-test, *: p< 0.05, **: p< 0.01, ***: p< 0.001) and the error bars show +/- SD of three biological replicates.

**Supplementary Fig. 6. The susceptible mutants *med16* and *cdk8* show the highest increase in metabolite levels and display unique differences in JA signaling in response to infection relative to Col-0 at the 72-hour time point. (A)** Log_2_-fold differences in levels of specific amino acids, lipids, and carbohydrates in infected *med16*, *med25*, and *cdk8* relative to Col-0 at the 72-hour time point. Only metabolites displaying differences in levels with p< 0.05 and a log_2_-FC > 1 for at least one mutant are included and the log_2_-fold values >0.5 for these metabolites are shown. Solid black lines represent a ±log_2_-FC of 0.5. **(B)** Metabolite levels of the JA derivates: JA-Ile, 12-OH-JA-Ile and MEJA, and the JA biosynthesis substrate α-linolenic acid in infected and mock-treated Col-0 and Mediator mutants at the 72-hour time point. **(C)** Heat map and hierarchical clustering of DEGs that belongs to the GO functional groups of jasmonic acid (GO:0009695, GO:0009753, GO:0009861, GO:0009867, GO:2000022, GO:0009694, GO:0009864, GO:0080141, GO:0032260, GO:0009871, GO:0071395, GO:0009868 and GO:0080140) in uninfected *med16*, *med18*, *med25* and *cdk8* relative to Col-0. **(D)** Relative mRNA expression levels of the JA-regulated *JAZ6* and *LOX2* genes in infected and mock-treated mutants and Col-0 at the 24-hour time point. Asterisks in **(B)** and **(D)** indicate pairwise significant differences between lines, treatments and time points as indicated by the brackets (Student's t-test, *: p< 0.05, **: p< 0.01, ***: p< 0.001). Error bars represent +/- SD of the four biological replicates in (B) and the three biological replicates in (D). Numbers without parentheses indicate fold change relative to mock-treated plants and numbers within parentheses indicate fold change of the comparisons indicated by the brackets.

**Supplementary** **Table S1.** Normalized peak values of metabolites detected by LC-TOF MS in uninfected (buffer) or infected (Pseudomonas) WT (Col-0), and arabidopsis Mediator subunit mutant lines at 24 and 72 hours p.i.

**Supplementary** **Table S2.** Ratios between levels of metabolites in infected and mock-treated mutants relative to infected and mock-treated Col-0 (control) at 24 and 72 hours p.i.

**Supplementary** **Table S3.** Division of metabolites into different categories.

**Supplementary** **Table S4.** Log_2_ fold changes of transcript levels in mutants relative to their respective wildtype controls.

**Supplementary** **Table S5.** Shared decreased metabolites, shared increased metabolites, unique decreased metabolites, unique increased metabolites and metabolites that change in opposite directions in mutants compared to Col-0 at control conditions (mock-treated) at both timepoints.

**Supplementary** **Table S6.** Metabolites that are decreased (log2-fold <1 and p-value <0.05) in Col-0 at 24 hours p.i., and their fold change in *med16*, *med18*, *med25* and *cdk8*.

**Supplementary** **Table S7.** Metabolites that are increased (log2-fold >1 and p-value <0.05) in Col-0 at 24 hours p.i., and their fold change in *med16*, *med18*, *med25* and *cdk8*.

**Supplementary** **Table S8.** Shared decreased metabolites, shared increased metabolites, unique decreased metabolites, unique increased metabolites and metabolites that change in opposite directions in mutants compared to Col-0 at 72h p.i.

**Supplementary** **Table S9.** Sequences for RT-qPCR primers.
