# Supplementary figures and images for: Pseudomonas syringae infectivity correlates to altered transcript and metabolite levels of Arabidopsis mediator mutants
Source: Sci Rep. 2024 Mar 21;14:6771. doi: 10.1038/s41598-024-57192-x (PMC10958028; doi:10.1038/s41598-024-57192-x)

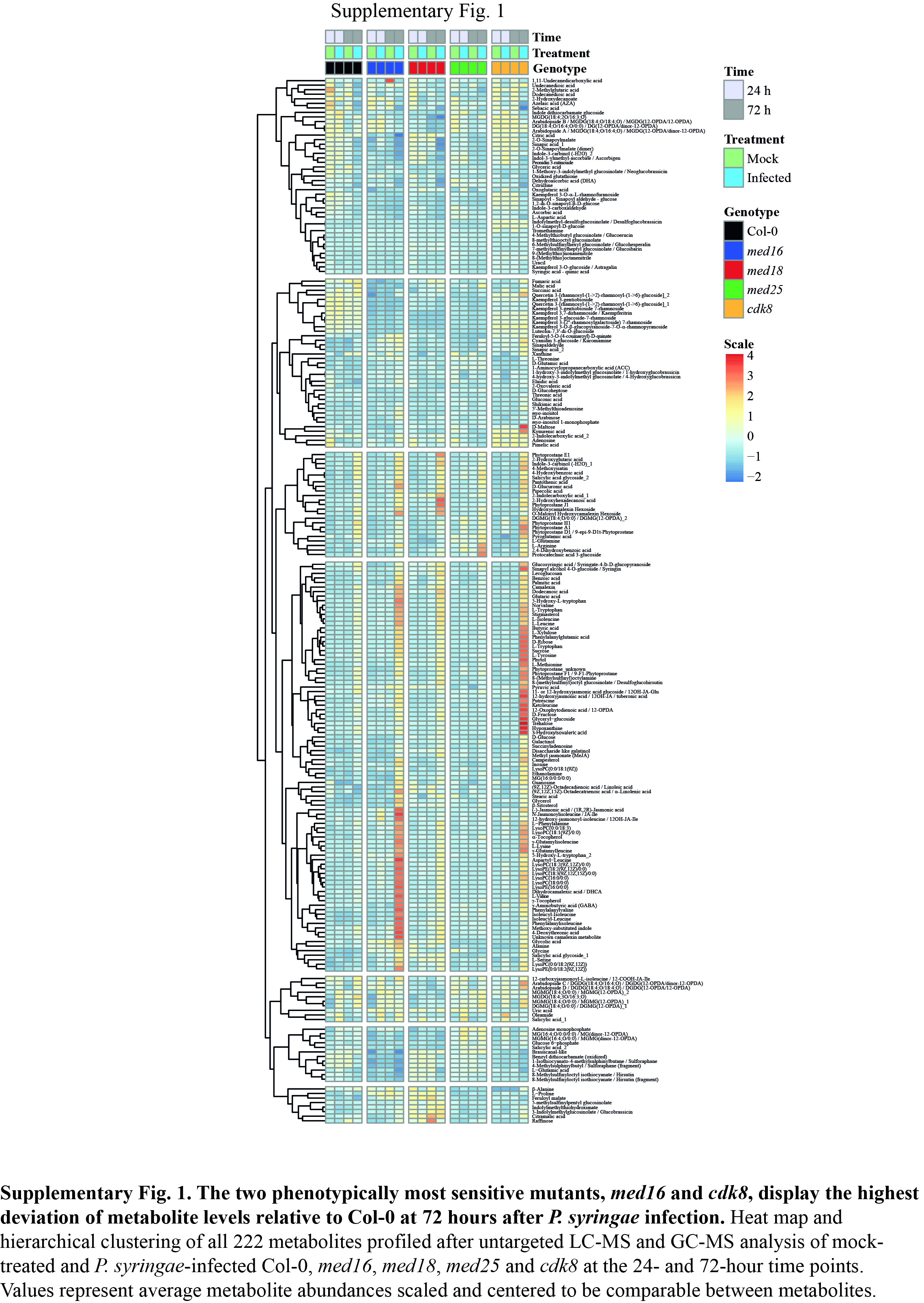

Supplement: Supplementary file 2 — Supplementary Figure 1. [file 41598_2024_57192_MOESM2_ESM.jpg]

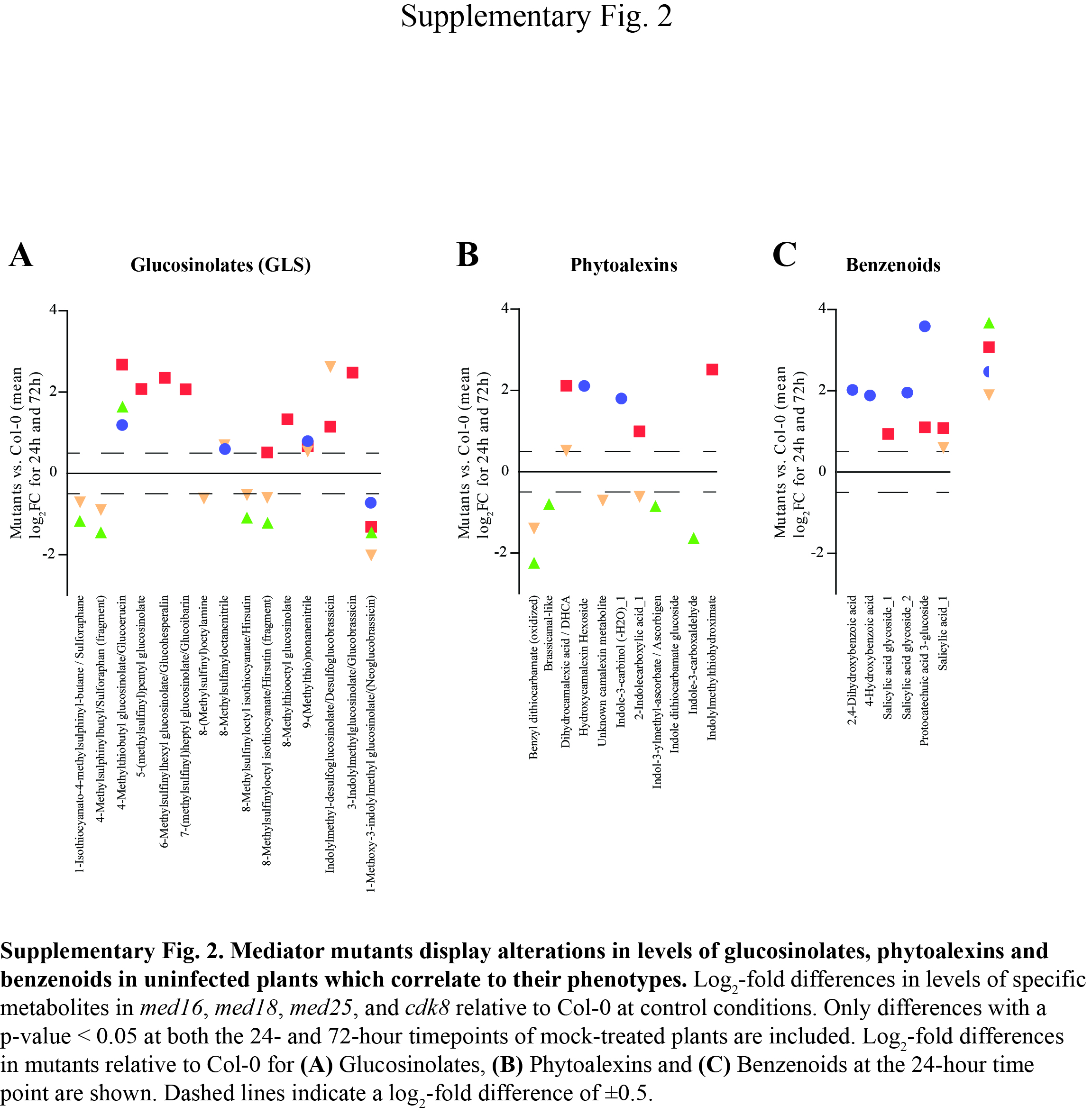

Supplement: Supplementary file 3 — Supplementary Figure 2. [file 41598_2024_57192_MOESM3_ESM.jpg]

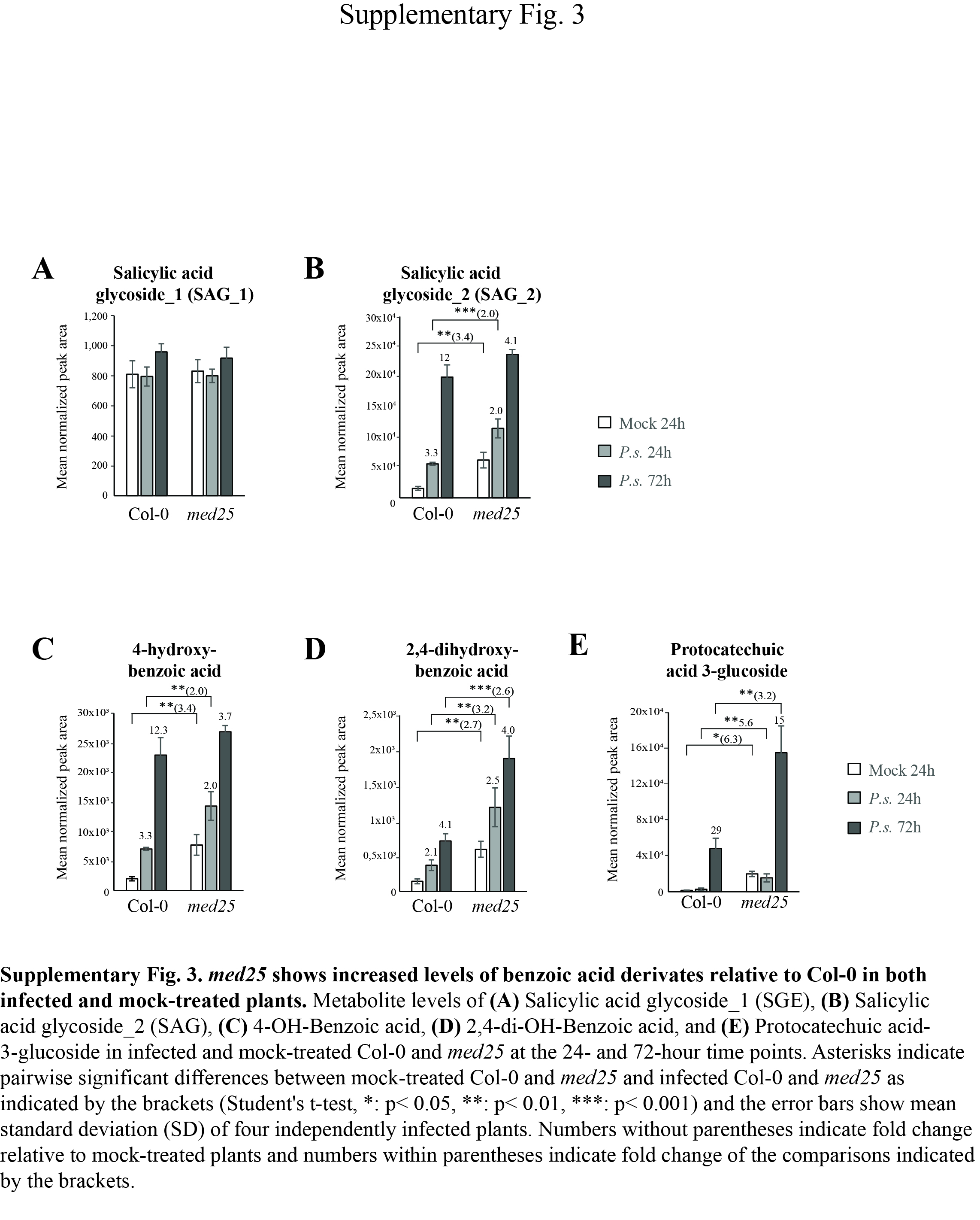

Supplement: Supplementary file 4 — Supplementary Figure 3. [file 41598_2024_57192_MOESM4_ESM.jpg]

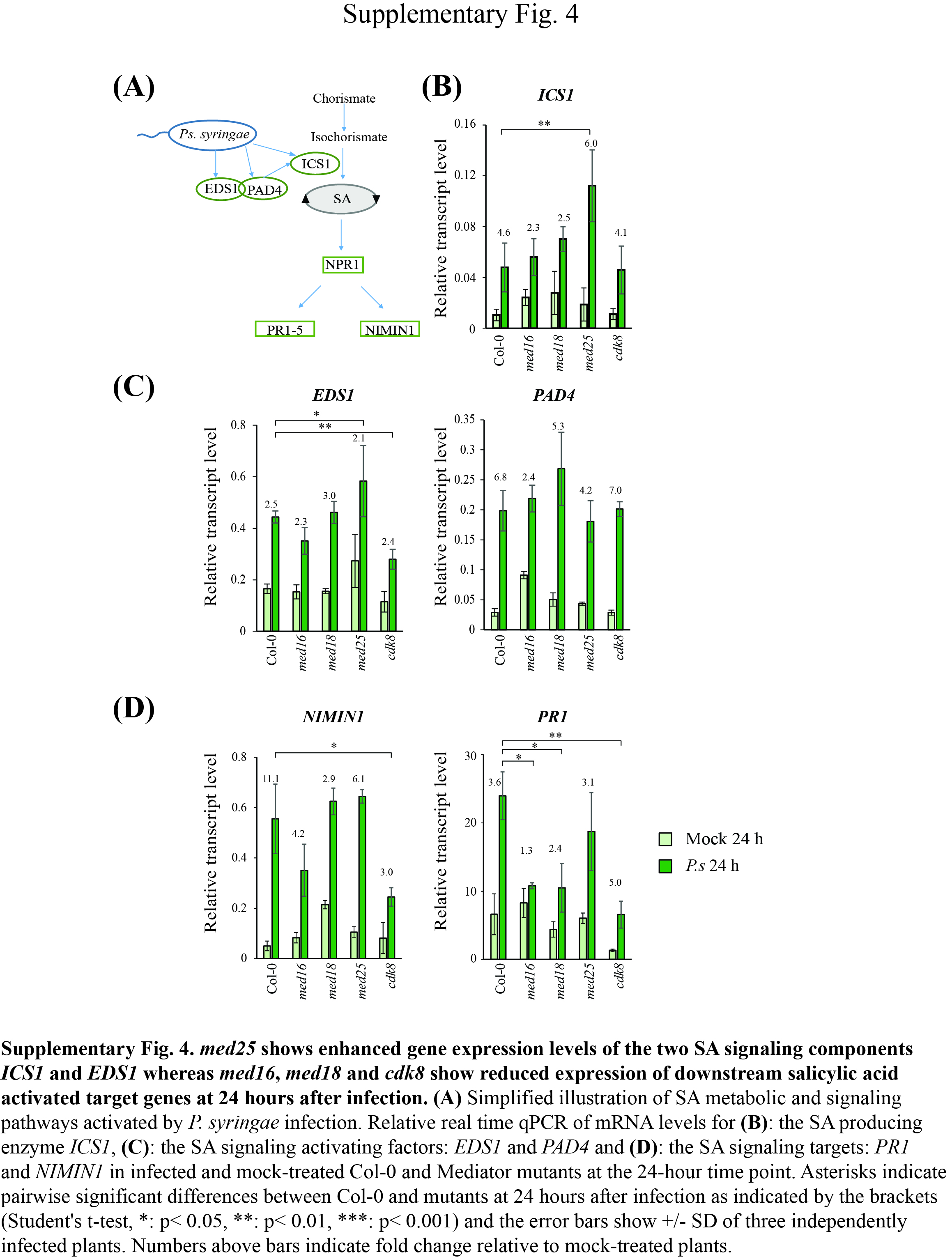

Supplement: Supplementary file 5 — Supplementary Figure 4. [file 41598_2024_57192_MOESM5_ESM.jpg]

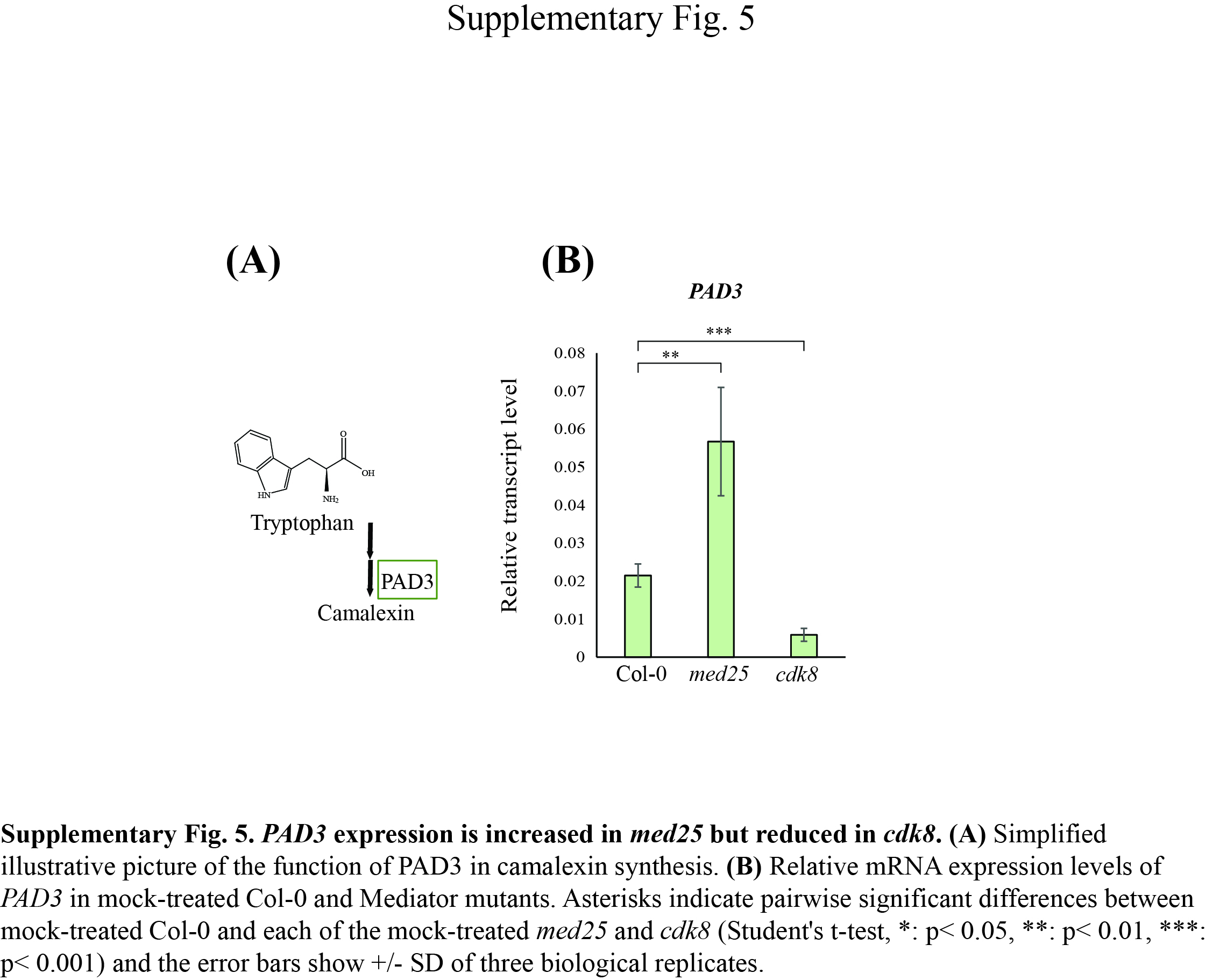

Supplement: Supplementary file 6 — Supplementary Figure 5. [file 41598_2024_57192_MOESM6_ESM.jpg]

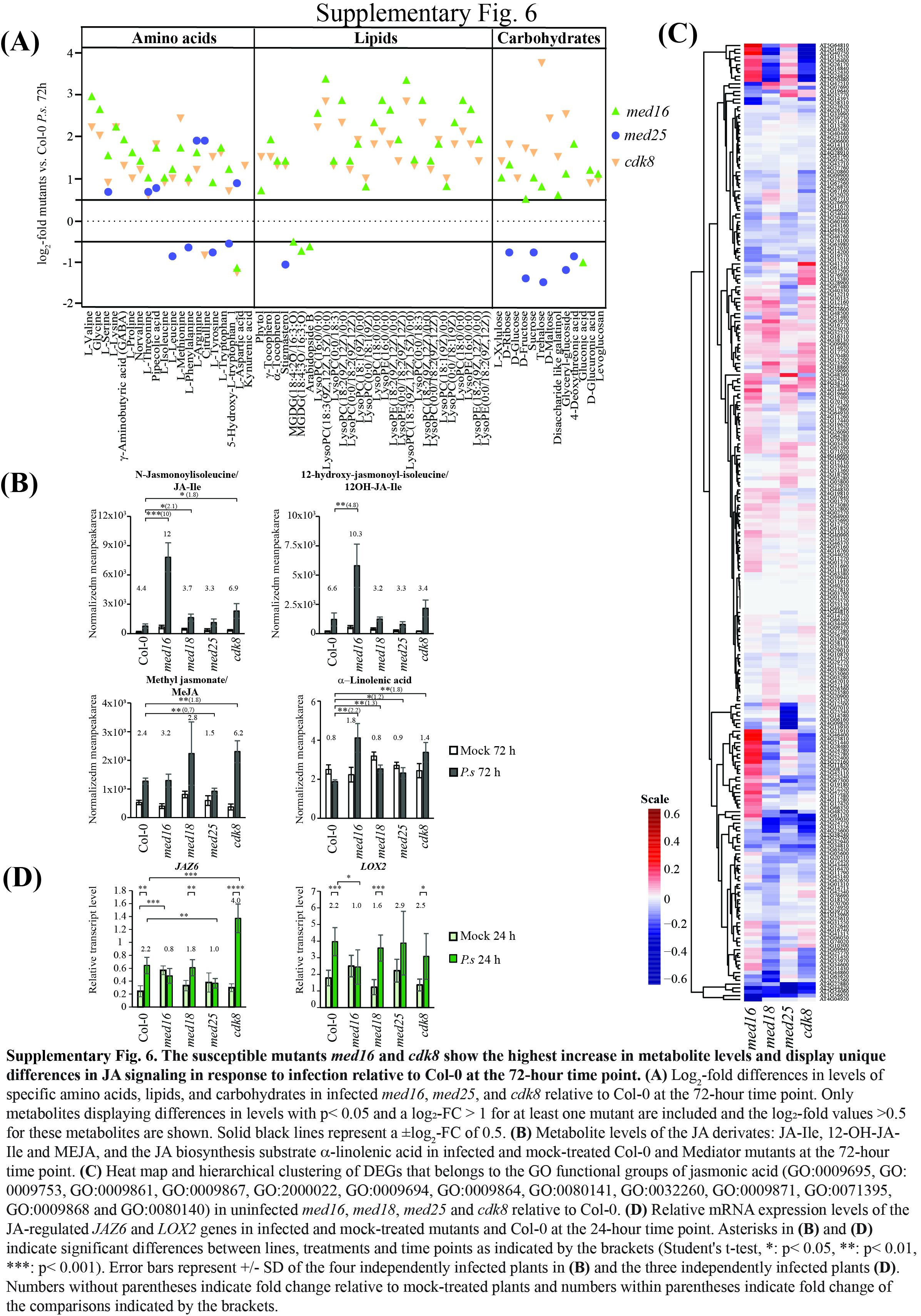

Supplement: Supplementary file 7 — Supplementary Figure 6. [file 41598_2024_57192_MOESM7_ESM.jpg]
